# PCSK9, A Promising Novel Target for Age-Related Cardiovascular Dysfunction

**DOI:** 10.1016/j.jacbts.2023.06.005

**Published:** 2023-09-13

**Authors:** Csaba Matyas, Eszter Trojnar, Suxian Zhao, Muhammad Arif, Partha Mukhopadhyay, Attila Kovacs, Alexandra Fabian, Marton Tokodi, Zsolt Bagyura, Bela Merkely, Laszlo Kohidai, Eszter Lajko, Angela Takacs, Yong He, Bin Gao, Janos Paloczi, Falk W. Lohoff, György Haskó, Wen-Xing Ding, Pal Pacher

**Affiliations:** a Laboratory of Cardiovascular Physiology and Tissue Injury, National Institute on Alcohol Abuse and Alcoholism, National Institutes of Health, Bethesda, Maryland, USA; b Department of Medical Imaging, Medical School, University of Pécs, Pécs, Hungary; c Heart and Vascular Center, Semmelweis University, Budapest, Hungary; d Department of Genetics, Cell and Immunobiology, Semmelweis University, Budapest, Hungary; e Laboratory of Liver Diseases, National Institute on Alcohol Abuse and Alcoholism, National Institutes of Health, Bethesda, Maryland, USA; f Section on Clinical Genomics and Experimental Therapeutics, National Institute on Alcohol Abuse and Alcoholism, National Institutes of Health, Bethesda, Maryland, USA; g Department of Anesthesiology, Columbia University, New York, New York, USA; h Department of Pharmacology, Toxicology and Therapeutics, University of Kansas Medical Center, Kansas City, Kansas, USA; i Department of Internal Medicine, University of Kansas Medical Center, Kansas City, Kansas, USA

**Keywords:** cardiovascular aging, heart failure, NASH, nonalcoholic steatohepatitis, PCSK9, transcriptomics

## Abstract

Cardiovascular diseases (CVDs) are the leading cause of death among elderly people. Proprotein convertase subtilisin/kexin type 9 (PCSK9) is an important regulator of cholesterol metabolism. Herein, we investigated the role of PCSK9 in age-related CVD. Both in humans and rats, blood PCSK9 level correlated positively with increasing age and the development of cardiovascular dysfunction. Age-related fatty degeneration of liver tissue positively correlated with serum PCSK9 levels in the rat model, while development of age-related nonalcoholic fatty liver disease correlated with cardiovascular functional impairment. Network analysis identified PCSK9 as an important factor in age-associated lipid alterations and it correlated positively with intima-media thickness, a clinical parameter of CVD risk. PCSK9 inhibition with alirocumab effectively reduced the CVD progression in aging rats, suggesting that PCSK9 plays an important role in cardiovascular aging.

## Highlights

• Age-associated development of NAFLD is associated with elevated liver/blood PCSK9 (a key regulator of cholesterol metabolism) level and correlates with the development of cardiovascular dysfunction.
• Increasing age is one of the strongest predictors of blood PCSK9 level, while blood PCSK9 level positively correlates with cardiovascular dysfunction and is an independent predictor of LV diastolic dysfunction.
• PCSK9 inhibition attenuates the progression of cardiovascular disease and NAFLD progression in aging animals.
• PCSK9 may emerge as a novel target and a potential biomarker for age-related cardiovascular disease.

## Visual Abstract

**Figure:**
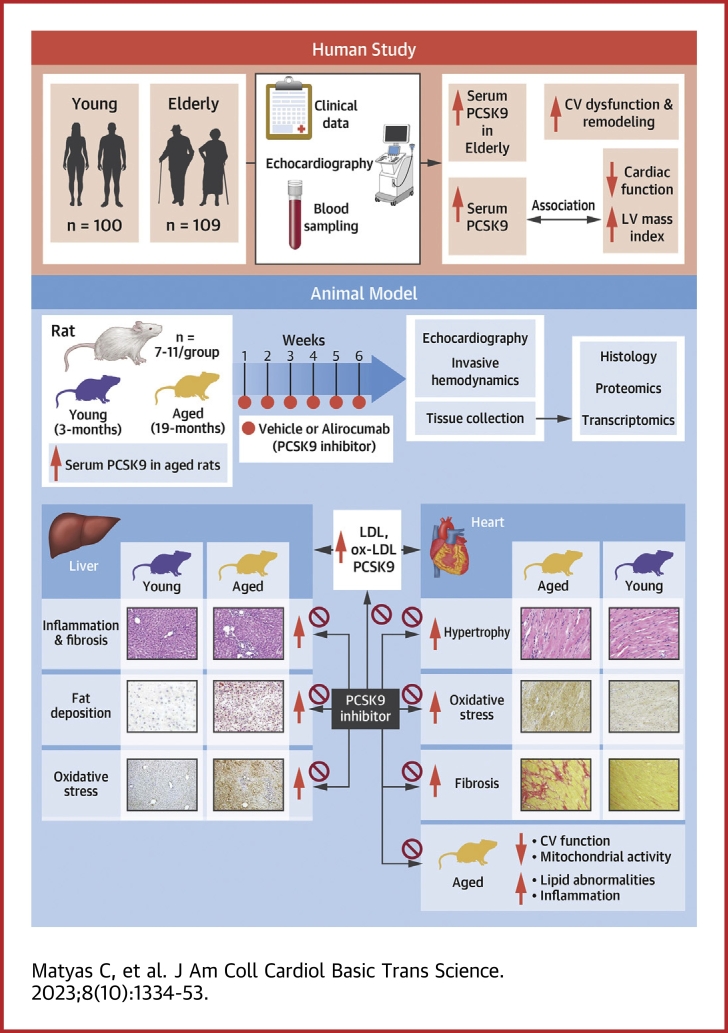

The average life expectancy is continuously increasing worldwide, and the population over 65 years of age is expected to double in the coming decades. The prevalence of cardiovascular and liver diseases, including vascular dysfunction, fatty liver, and heart failure, increases with age^1^^,^^2^ and is expected to further increase over the next decades.

Cardiovascular aging is characterized by atherosclerosis and the remodeling of the myocardium such as hypertrophy and fibrosis, and will eventually culminate in the development of heart failure,^3^ while hallmarks of liver aging are fatty degeneration, fibrosis, and inflammation.^2^ The prevalence of nonalcoholic fatty liver disease (NAFLD) increases with age,^4^ and NAFLD is associated with increased cardiovascular risk, including heart failure.^4^^,^^5^ Major pathophysiological events in age-related cardiovascular disorders include, but are not limited to, oxidative/nitrative stress^6^ and mitochondrial dysfunction.^7^^,^^8^ Ultimately, these pathways culminate in cardiovascular dysfunction and remodeling of the cardiac tissue with characteristic changes in the extracellular matrix and finally with the development of myocardial hypertrophy and fibrosis.^9^ Despite the need for effective drug therapies to prevent or treat aging-associated heart failure, therapeutic options are largely limited for this condition, especially taking the prevalence of heart failure with preserved ejection fraction into account.

Aging is associated with dysregulation of cholesterol metabolism.^10^ Proprotein convertase subtilisin/kexin type 9 (PCSK9) is a member of a class of proteinase K–like serine proteases first described in cerebellar cells as a regulator of apoptosis.^11^ Later, PCSK9 was identified as one of the most important regulators of cholesterol metabolism via the regulation of the expression of low-density lipoprotein receptor (LDLR) in the liver, where it binds to liver LDLRs, thereby promoting their degradation.^12^ As a result, low-density lipoprotein (LDL) cholesterol uptake is reduced by the lower number of available LDLRs, which will eventually lead to hypercholesterolemia.^13^ PCSK9 is expressed in different organs, and while it is most abundant in the liver, it is expressed at lower levels in the heart, too.^12^ Levels of PCSK9 depend on many factors including, for example, age, sex, diurnal rhythm, nutrition, and comorbidities.^14^ PCSK9 expression is regulated by many factors, with one of the most important among them being the sterol regulatory element-binding proteins (SREBPs).^15^ SREBPs are activated by oxidative stress,^16^ during inflammation,^17^ or by aging,^17^, ^18^, ^19^ contributing to altered lipid metabolism and steatosis.^17^ Moreover, circulating PCSK9 levels are associated with hepatic fat content and NAFLD severity.^20^ PCSK9 was recently identified as a potential therapeutic target to treat hypercholesterolemia, first in targeted patient populations of familial hypercholesterolemia and followed by cardiovascular diseases, in which recommended cholesterol levels were not met or cardiovascular risk remained high despite optimal drug therapy.^14^^,^^21^

Antibodies, such as alirocumab,^22^ targeted against circulating PCSK9 are a novel class of lipid-lowering agents, which, in combination with or without statins, have been shown to successfully reduce blood cholesterol levels and the incidence of cardiovascular events, relative risk, and mortality of cardiovascular diseases.^21^, ^22^, ^23^, ^24^, ^25^ Recently, a number of studies investigated the pleiotropic effects of PCSK9 inhibitors beyond their lipid-lowering properties. PCSK9 stimulates oxidized LDL formation^26^ and participates in a bidirectional crosstalk with reactive oxygen species formation,^27^ leads to endothelial activation/damage,^28^ and is a critical immune response regulator in sepsis.^29^ On the other hand, PCSK9 inhibition proved to have antioxidant effects in alcohol-induced liver disease^30^ or in H_2_O_2_-induced oxidative damage,^31^ was able to reduce oxidized LDL–induced myocardial injury,^32^ and was recently shown to reduce mortality or serious complications and inflammatory response in COVID-19 patients.^33^

Although representing a major group of Western societies, elderly people are often underrepresented in clinical trials of lipid-lowering therapies.^34^

Despite available data about the beneficial effects of PCSK9 inhibitor antibodies in cardiovascular diseases and hypercholesterolemia, specific data about PCSK9 metabolism and the effects of PCSK9 inhibitors on cardiovascular health in elderly are limited. On the other hand, plasma PCSK9 levels have been shown to correlate with age and multiple metabolic parameters in youth^35^; however, data about elderly are largely limited.

Thus, we aimed at investigating the relationship between PCSK9 levels, age-related hepatic changes, and cardiovascular dysfunction in aging and the effect of the PCSK9 inhibitor alirocumab on age-related cardiac decline in an experimental setting.

## Methods

### Human study

#### Patient population

**Figure 1:**
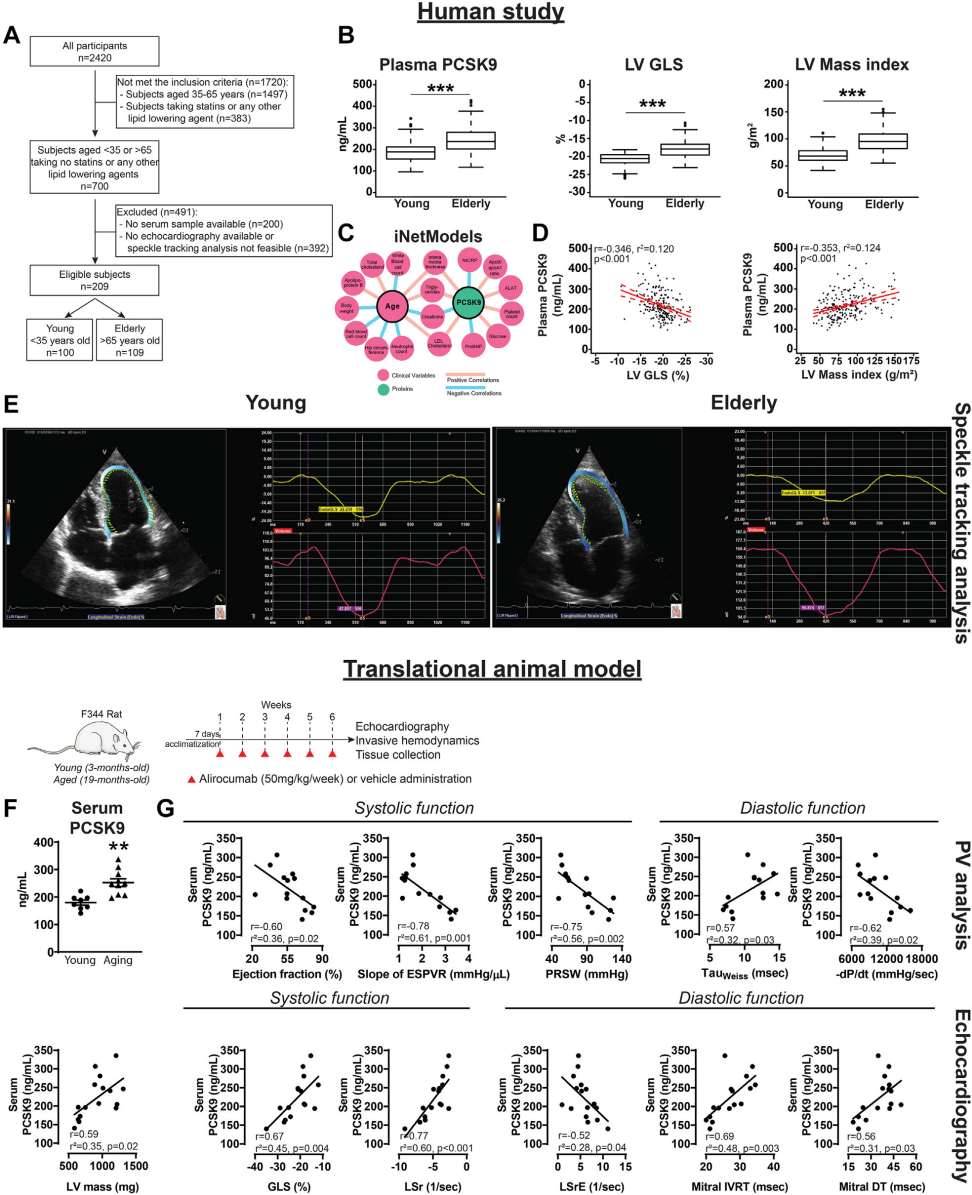
Increased PCSK9 Levels Are Associated With Age-Related Cardiovascular Dysfunction Human study (top). (A) Study design. (B) Box plots for plasma proprotein convertase subtilisin/kexin type 9 (PCSK9) levels, left ventricular (LV) global longitudinal strain (GLS), and LV mass index values. Statistics: for PCSK9 and GLS, Mann-Whitney *U* test; for LV mass index, Student’s unpaired *t* test. (C) Network from the iNetModels database. (D) Correlation of PCSK9 levels with LV GLS and LV mass index. (E) Representative speckle-tracking strain analyses from the study groups. Translational animal model (bottom, with study design). (F) Serum PCSK9 levels (Student’s unpaired *t* test). Scattered dot plots show mean ± SEM. Correlation of PCSK9 levels with functional parameters derived from (G) pressure-volume (PV) analysis and echocardiography. ∗*P <* .05, ∗∗*P <* .01, ∗∗∗*P <* .001. −dP/dt = minimal slope of maximal rate of left ventricular pressure rise; DT = deceleration time; ESPVR = end-systolic pressure-volume relationship; IVRT = isovolumic relaxation time; LSr = longitudinal strain rate; LSrE = longitudinal strain rate E-wave; PRSW = preload recruitable stroke work; Tau_Weiss_ = left ventricular diastolic time constant.

The current study was carried out from a selected patient population of a voluntary, longitudinal, population-based screening program (detailed description in the Supplemental Appendix). The study was approved by the Hungarian Scientific and Research Ethics Committee of the Medical Research Council (Approval No.: 8224-0/2011/EKU [265/PI/11]). Procedures followed the ethical standards of the responsible committee on human experimentation (institutional and national) and the Helsinki Declaration of 1975 (fifth revision). Written informed consent was obtained from all study participants. We aimed to define 2 age categories: a young group with subjects younger than 35 years of age and an elderly group older than 65 years of age. Exclusion criteria were: 1) subjects taking statins or any other lipid-lowering agents; 2) no serum samples available; 3) no echocardiographic recordings available; and 4) no apical 4-chamber view recordings available appropriate for speckle-tracking analysis based on the criteria shown in Figure 1A. Further details are available in the Supplemental Appendix.

#### Echocardiography and speckle-tracking echocardiography

The details of the echocardiography are described in the Supplemental Appendix.

#### Determination of plasma PCSK9 levels and network analysis of PCSK9 and aging

The determination of plasma PCSK9 levels and network analysis of PCSK9 and aging from a separate multiomics database iNetModels, and results of human bulk, single-nuclei, and spatial transcriptomics data are described in the Supplemental Appendix.

### Animal study

#### Treatment protocol

Male F344/DuCrl (Fisher) young (3 months of age) and aging (19 months of age) rats were obtained from the National Institute on Aging. The study was reviewed and approved by the Institutional Animal Care and Use Committee of the National Institute on Alcohol Abuse and Alcoholism and conformed to the National Institutes of Health guidelines on animal experiments (Guide for the Care and Use of Laboratory Animals prepared by the National Academy of Sciences and published by the National Institutes of Health [publication 86-23, revised 1985]). The housing of rats and treatment protocol with PCSK9 inhibitor alirocumab and sample harvesting are described in the Supplemental Appendix.

#### Echocardiography and speckle-tracking myocardial strain analysis and invasive pressure-volume analysis

Echocardiography and speckle-tracking myocardial strain analysis and invasive pressure-volume analysis in rats were performed as detailed in the Supplemental Appendix.

#### Biochemistry

Measurement of serum cholesterol, oxidized LDL, and B-type natriuretic peptide; determination of serum and liver PCSK9 levels; and liver triglyceride content are described in the Supplemental Appendix.

Western blotting; measurements of myocardial PARP1, caspase 3, and mitochondrial complex activities; RNA isolation and quantitative real-time polymerase chain reaction; messenger RNA sequencing; and histology are described in the Supplemental Appendix.

The raw RNA-sequencing files can be accessed via the Gene Expression Omnibus database with accession number GSE225859.

### Statistical analysis

Normality of distribution was tested by the Shapiro-Wilk test. Continuous variables are expressed as mean ± SD, mean ± SEM, or median (IQR) as noted, whereas categorical variables are reported as frequency and percentage. Groups were compared with unpaired Student’s *t* test or Mann-Whitney *U* test for continuous variables and chi-square or Fisher exact test for categorical variables, as appropriate. One-way analysis of variance was carried out with Tukey’s post hoc test for multiple pairwise comparisons. Pearson’s correlation coefficient (*R*) was computed to assess the association between continuous variables. We used univariable and multivariable linear regression analysis to investigate the associations of plasma PCSK9 levels and the ratio between early mitral inflow velocity and mitral annular early diastolic velocity (E/e′) with age, sex, body mass index (BMI), and relevant cardiovascular risk factors in humans. In the univariable and multivariable linear regression models, the associations between the dependent and independent variables are reported using regression coefficients (β) with SE. The results of the *F* test and the adjusted coefficient of determination (*R*^2^) are also reported for each multivariable model. Only variables exhibiting a *P* value of <0.1 in univariable models were considered in the multivariable analysis. Collinearity was tested using the variance inflation factor (excessive if variance inflation factor >3). A 2-sided *P* value of <0.05 was considered statistically significant.

To perform differential expression analysis for the transcriptomic data, the raw count files from HISAT2 were used as an input to the DESeq2 package in R version 4.0.3 (R Foundation for Statistical Computing), and genes with *P* value <0.05 were considered as significantly differentially expressed genes. The gene-level statistics (log2 fold changes and *P* value) from DESeq2 were then used to perform functional analysis, together with the gene-set collection from the Kyoto Encyclopedia of Genes and Genomes and Gene Ontology downloaded from the Enrichr library. The functional analyses were performed using the PIANO package in R. The Benjamin-Hochberg false discovery rate was used for the adjustment. Kyoto Encyclopedia of Genes and Genomes or Gene Ontology terms with false discovery rate <0.05 were considered significant. Visualization of the results was done using Seaborn package in Python 3.7 (Python Software Foundation).

Statistical analysis was performed in R version 4.0.3 or in GraphPad Prism 7.0 for Windows (GraphPad Software). Results with *P* < 0.05 were accepted as statistically significant.

## Results

### Human study

#### Basic characteristics of the study groups

According to our predefined inclusion and exclusion criteria (Figure 1A), 100 patients were included in the young group and 109 in the elderly group of the final study population. Both groups were characterized by a modest female predominance (Table 1). The subjects in the young group had higher height, lower weight, and correspondingly lower BMI. The prevalence of hypertension, hyperlipidemia, and diabetes mellitus was significantly higher in the elderly group. There were more current smokers among the young. Elderly individuals presented with higher serum levels of total cholesterol, triglyceride, and LDL cholesterol. High-density lipoprotein levels were similar between the 2 groups (Table 1). The medication list for the study groups is listed in Supplemental Table 1.

**Table 1:** Clinical Characteristics of the Study Groups

	Young (n = 100)	Elderly (n = 109)	*P* Value
Age, y	29 (25-33)	72 (68-76)	<0.001
Male	49 (49)	45 (41)	0.32
Height, cm	172 (167-180)	160 (156-169)	<0.001
Weight, kg	69 (60-84)	77 (67-87)	0.046
BMI, kg/m^2^	23.0 (21.1-26.0)	28.0 (25.7-31.0)	<0.001
BSA, m^2^	1.8 (1.7-2.0)	1.8 (1.7-2.0)	0.84
Hypertension	9 (9)	66 (61)	<0.001
Hyperlipidemia	6 (6)	25 (23)	<0.001
Diabetes mellitus	1 (1)	20 (18)	<0.001
Former smokers	51 (51)	41 (38)	0.071
Current smokers	32 (32)	5 (5)	<0.001
Creatinine, μmol/L	75 (61-85)	80 (68-91)	0.020
GFR, mL/min/1.73 m^2^	98 (83-108)	72 (59-80)	<0.001
Glucose, mmol/L	5.2 (4.9-5.7)	5.7 (5.3-6.5)	<0.001
HbA_1c_ (n = 204), %	5.3 (5.1-5.5)	5.8 (5.5-6.1)	<0.001
Triglyceride, mmol/L	1.2 (0.8-1.8)	2.0 (1.5-2.9)	<0.001
Total cholesterol, mmol/L	4.9 ± 0.9	5.8 ± 1.0	<0.001
LDL-C (n = 199), mmol/L	2.8 (2.3-3.3)	3.6 (3.1-4.1)	<0.001
HDL-C, mmol/L	1.5 (1.2-1.8)	1.4 (1.2-1.7)	0.17
ASAT, U/L	18 (16-21)	19 (16-23)	0.21
ALAT, U/L	16.5 (13.0-23.0)	16.0 (13.0-21.0)	0.92
GGT, U/L	13 (10-22)	21 (14-31)	<0.001
ALP, U/L	63 (53-78)	74 (63-89)	<0.001
hs-CRP (n = 208), mg/L	1.0 (0.5-2.8)	2.2 (1.1-4.9)	<0.001
NT-proBNP (n = 205), pg/mL	33 (15-60)	99 (69-209)	<0.001
CIMT (n = 174), mm	0.6 (0.6-0.6)	0.9 (0.9-1.0)	<0.001
PCSK9, ng/mL	188 (156-211)	236 (202-279)	<0.001

Values are median (IQR), n (%), or mean ± SD. The characteristics of the 2 groups were compared using unpaired Student’s *t* test or Mann-Whitney *U* test for continuous variables and chi-square or Fisher exact test for categorical variables, as appropriate.

ALAT = alanine aminotransferase; ALP = alkaline phosphatase; ASAT = aspartate aminotransferase; BMI = body mass index; BSA = body surface area; CIMT = maximal carotid intima-media thickness; GFR = glomerular filtration rate; GGT = gamma-glutamyltransferase; HbA_1c_ = glycosylated hemoglobin; HDL-C = high-density lipoprotein cholesterol; hs-CRP = high-sensitivity C-reactive protein; LDL-C = low-density lipoprotein cholesterol; NT-proBNP = N-terminal pro–B-type natriuretic peptide; PCSK9 = proprotein convertase subtilisin/kexin type 9.

#### Echocardiography data of the study groups

Two-dimensional and speckle-tracking echocardiographic data are summarized in Table 2. Higher values of interventricular septal, left ventricular (LV) posterior wall thicknesses, and LV mass index (Figure 1B) were present in the elderly group, while LV end-diastolic volume did not differ. However, LV systolic function was significantly lower based on the reduced values of ejection fraction and global longitudinal strain (GLS) as well (Figure 1B). The ratio of early mitral inflow velocity to late mitral inflow velocity (E/A) was lower, E-wave deceleration time was longer, average early diastolic mitral annular velocity (e′) was lower, and E/e′ values were higher, while left atrial peak left atrial longitudinal strain (LA PALS) was reduced in the elderly, pointing at LV diastolic dysfunction. Correspondingly, left atrial volume was significantly higher. Concerning the right heart, the right ventricle was larger in the elderly group, but the right atrial volume and right ventricular systolic function (quantified by tricuspid annular plane systolic excursion [TAPSE]) were similar. The peak velocity of the tricuspid regurgitation jet was higher among the elderly individuals, suggesting elevated pulmonary artery systolic pressure (Table 2).

**Table 2:** Echocardiographic Characteristics of the Study Groups

	Young (n = 100)	Elderly (n = 109)	*P* Value
IVSd (n = 202), mm	8 (8–9)	13 (11–14)	<0.001
LVIDd (n = 202), mm	48 ± 4	47 ± 5	0.059
LVPWd (n = 202), mm	7 (7–8)	9 (8–10)	<0.001
LVMi (n = 202), g/m^2^	68 (61–78)	95 (82–109)	<0.001
LVEDVi, mL/m^2^	70 (63–82)	76 (66–88)	0.048
LVESVi, mL/m^2^	33 (28–39)	38 (32–45)	<0.001
LVSVi, mL/m^2^	38 (33–44)	38 (33–43)	0.78
LVEF, %	54 (48–59)	49 (47–53)	<0.001
LVGLS, %	−20.5 (−21.7 to −19.5)	−17.9 (−19.6 to −16.6)	<0.001
LAVi, mL/m^2^	27 (23–31)	33 (27–43)	<0.001
PALS, %	−31.4 ± 4.7	−26.5 ± 5.0	<0.001
RAVi, mL/m^2^	25 (21–30)	26 (21–33)	0.75
RV basal diameter, mm	31 ± 4	33 ± 5	<0.001
TAPSE (n = 194), mm	23 (21–26)	22 (20–25)	0.25
AV peak velocity (n = 203), m/s	1.2 (1.1–1.3)	1.4 (1.2–1.5)	0.003
E (n = 205), cm/s	87 (76–95)	65 (54–80)	<0.001
A (n = 203), cm/s	53 (44–60)	81 (68–92)	<0.001
E/A (n = 203)	1.6 (1.4–1.9)	0.8 (0.7–1.0)	<0.001
DT (n = 205), ms	165 (138–193)	225 (196–279)	<0.001
Septal e′ (n = 202), cm/s	17 (15–19)	9 (6–11)	<0.001
Lateral e′ (n = 202), cm/s	13 (11–14)	7 (6–8)	<0.001
Average E/e′ (n = 198)	6.0 (5.1–6.7)	8.6 (7.6–10.7)	<0.001
TR peak velocity (n = 117), m/s	1.9 ± 0.2	2.2 ± 0.4	<0.001

Values are median (IQR) or mean ± SD. The characteristics of the 2 groups were compared using unpaired Student’s *t* test or Mann-Whitney *U* test, as appropriate.

A = late mitral inflow velocity; AV = aortic valve; DT = E-wave deceleration time; E = early mitral inflow velocity; e′ = early diastolic mitral annular velocity; IVSd = thickness of the interventricular septum at end-diastole; LAVi = left atrial volume index; LVEDVi = left ventricular end-diastolic volume index; LVEF = left ventricular ejection fraction; LVESVi = left ventricular end-systolic volume index; LVGLS = left ventricular global longitudinal strain; LVIDd = left ventricular internal diameter at end-diastole; LVMi = left ventricular mass index; LVPWd = thickness of the left ventricular posterior wall at end-diastole; LVSVi = left ventricular stroke volume index; PALS = peak left atrial longitudinal strain; RAVi = right atrial volume index; RV = right ventricular; TAPSE = tricuspid annular plane systolic excursion; TR = tricuspid regurgitation.

#### Increased PCSK9 levels were associated with age-related cardiovascular dysfunction

Plasma PCSK9 levels were significantly higher in the elderly group (Figure 1B). The network from iNetModels database showed that PCSK9 and age both positively correlated with serum triglyceride and LDL cholesterol, and age positively correlated with total cholesterol level (Figure 1C). In the pooled population of the Budakalász Study, higher PCSK9 levels were associated with higher LV mass index and reduced LV GLS (Figure 1D). In a multivariable linear regression model, age, sex, BMI, and hyperlipidemia were independent predictors of plasma PCSK9 levels (Table 3). In another model, PCSK9 level and hypertension were found to be independent predictors of E/e′ (ie, diastolic dysfunction) (Table 4). Representative echocardiographic apical 4-chamber view images and corresponding speckle-tracking analysis to determine GLS in the young and the elderly are shown in Figure 1E. In a separate analysis, PCSK9 gene expression levels were very low in heart compared with liver in the human RNA sequencing data obtained from the Genotype-Tissue Expression Project Portal (Supplemental Table 2). Cardiac gene expression levels of PCSK9 in human and rat hearts showed very low levels of PCSK9 expression in hearts (Supplemental Table 3). Protein levels of PCSK9 were higher in aging hearts (Supplemental Table 3). On the other hand, single-nuclei transcriptomics showed that PCSK9 was only expressed in <0.07%, while troponin T, as a reference gene, was expressed in almost all of the ventricular cardiomyocyte cells. Spatial transcriptomics data also showed that PCSK9 level in heart was very low compared with troponin T (Supplemental Figure 1).

**Table 3:** Univariable and Multivariable Linear Regression Analysis to Determine the Predictors of Plasma PCSK9 Levels (n = 209)

	Univariable		Multivariable		
	**β** (SE)	*P* Value	**β** (SE)	*P* Value	VIF
Age	1.259 (0.169)	<0.001	1.038 (0.208)	<0.001	1.754
Male	−29.677 (8.374)	<0.001	−26.711 (7.265)	<0.001	1.042
BMI	4.232 (0.808)	<0.001	1.920 (0.857)	0.026	1.457
Hypertension	31.907 (8.665)	<0.001	−7.160 (9.179)	0.43	1.546
Diabetes mellitus	23.612 (14.176)	0.097	−7.925 (12.821)	0.53	1.185
Hyperlipidemia	62.300 (11.268)	<0.001	43.238 (10.509)	<0.001	1.113
Former smoker	−4.136 (8.638)	0.63	—	—	—
Current smoker	−21.305 (11.142)	0.057	13.110 (10.085)	0.195	1.182

Only variables with a *P* of <0.10 were included in the multivariable model. Multivariable model: *F*_7,201_ = 14.7; *P <* 0.001; adjusted *R*^2^ = 0.316.

VIF = variance inflation factor; other abbreviations as in Tables 1 and 2.

**Table 4:** Univariable and Multivariable Linear Regression Analysis to Determine the Predictors of Average E/e′ (n = 198)

	Univariable		Multivariable		
	**β** (SE)	*P* Value	**β** (SE)	*P* Value	VIF
PCSK9	0.015 (0.004)	<0.001	0.010 (0.004)	0.006	1.350
Male	−0.942 (0.418)	0.025	−0.698 (0.386)	0.072	1.083
BMI	0.152 (0.041)	<0.001	0.018 (0.044)	0.67	1.388
Hypertension	2.603 (0.399)	<0.001	2.089 (0.439)	<0.001	1.296
Diabetes mellitus	2.138 (0.683)	0.002	0.976 (0.659)	0.140	1.152
Hyperlipidemia	1.204 (0.582)	0.040	−0.311 (0.572)	0.58	1.228

E/e′ was available for only 198 (95%) patients in our study cohort. Multivariable model: *F*_6,191_ = 10.7; *P <* 0.001; adjusted *R*^2^ = 0.227.

Abbreviations as in Tables 1, 2, and 3.

### Animal study

#### Serum PCSK9 levels correlated with cardiac dysfunction

Serum PCSK9 levels were significantly increased in the aging animals and its level correlated positively with LV mass values (Figure 1F). Serum PCSK9 levels correlated with the development of systolic dysfunction (ejection fraction, slope of end-systolic pressure-volume relationship [ESPVR], preload recruitable stroke work [PRSW], GLS, longitudinal systolic strain rate [LSr]) and diastolic (left ventricular diastolic time constant [Tau_Weiss_], minimal slope of maximal rate of left ventricular pressure rise [−dP/dt], longitudinal early systolic strain rate [LSrE], mitral isovolumic relaxation time [IVRT], mitral deceleration time [DT]) (Figure 1G). Of note, PCSK9 protein and gene expression values were very low in the rat heart in comparison with the liver, which was further supported by independent mouse data (Supplemental Table 2).

#### Aging led to characteristic changes in the liver

**Figure 2:**
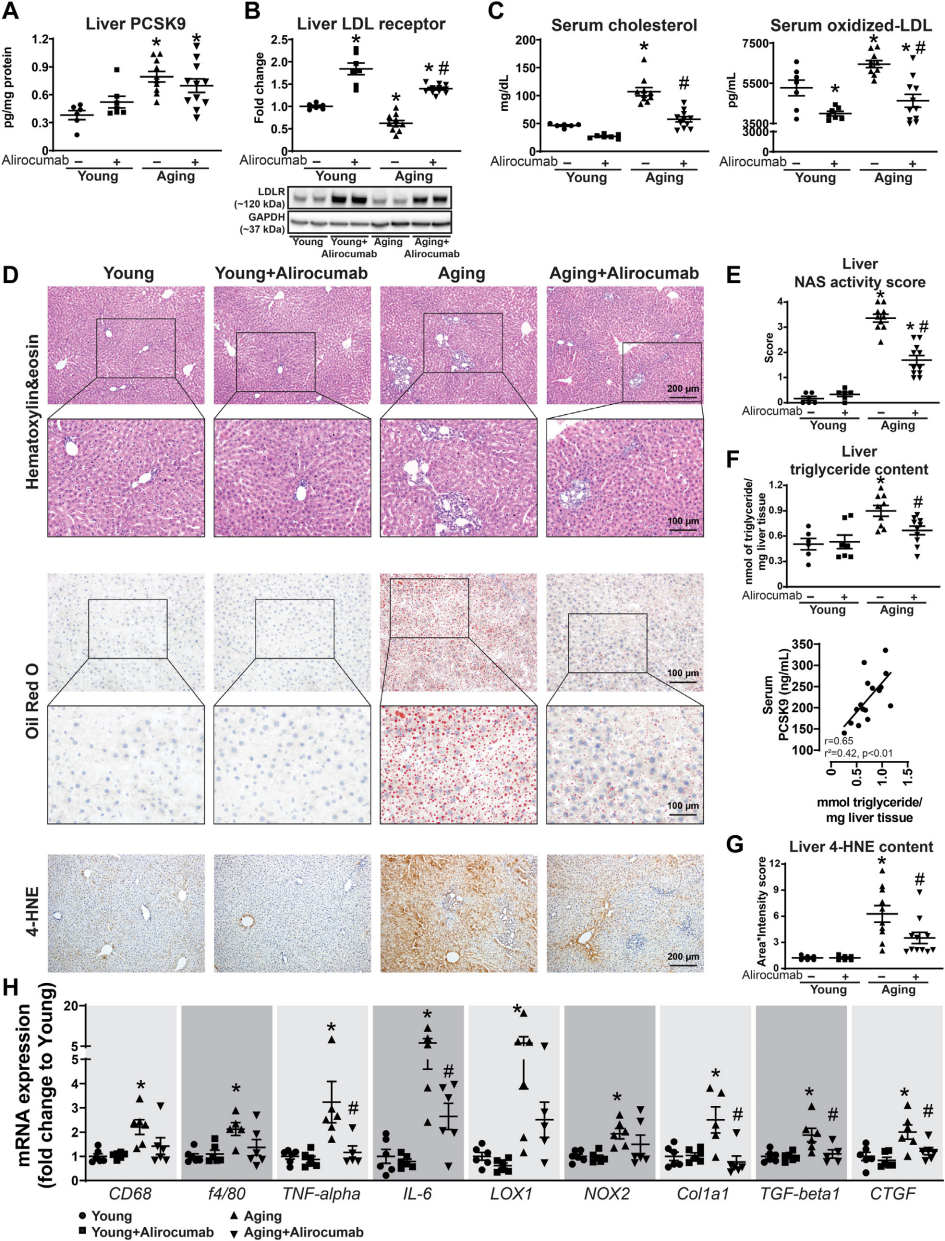
Increased PCSK9 Levels Are Associated With Age-Related Liver Changes (A) Liver proprotein convertase subtilisin/kexin type 9 (PCSK9) levels. (B) Liver low-density lipoprotein receptor (LDLR) levels and representative Western blot. (C) Serum cholesterol and oxidized low-density lipoprotein (LDL) levels. (D) Liver histology, liver Oil red O staining, and 4-hydroxynonenal (4-HNE) immunohistochemistry. (E) Nonalcoholic fatty liver disease activity score (NAS). (F) Liver triglyceride content and its correlation with serum PCSK9 levels. (G) Liver 4-HNE scoring. (H) Gene expression of CD68, f4/80, tumor necrosis factor (TNF)-alpha, interleukin (IL)-6, oxidized LDL receptor 1 (LOX1), NADPH oxidase 2 (NOX2), collagen 1a1 (Col1a1), transforming growth factor (TGF)-beta1, and connective tissue growth factor (CTGF). ∗*P <* 0.05 vs young; #*P <* 0.05 vs aging. One-way analysis of variance with Tukey’s post hoc test. Scattered dot plots, show mean ± SEM. mRNA = messenger RNA.

PCSK9 levels were significantly increased in the aging liver (Figure 2A). Liver LDLR expression was decreased in the aging animals, which was in line with the observed increase in serum total cholesterol and oxidized LDL levels (Figures 2B and 2C). Alirocumab significantly increased the LDLR expression and led to a decrease in total cholesterol and oxidized LDL levels (Figures 2B and 2C). The aging liver was characterized by fatty, inflammatory, and fibrotic changes (NAFLD activity score [NAS]) (Figures 2D to 2F), along with increased oxidative stress (Figure 2G). Gene expression studies showed overexpression of inflammatory, oxidative stress, and fibrosis-related genes (Figure 2H). Alirocumab effectively reduced the fat accumulation and inflammatory and fibrotic changes in the aging liver (Figures 2D to 2H). Of note, serum PCSK9 levels positively correlated with liver fat content (Figure 2F). The drug treatment had no adverse effects on the liver, represented by unchanged aspartate and alanine transaminase levels in the rats (Supplemental Figure 2).

#### NAFLD development correlated with cardiovascular functional impairment

**Figure 3:**
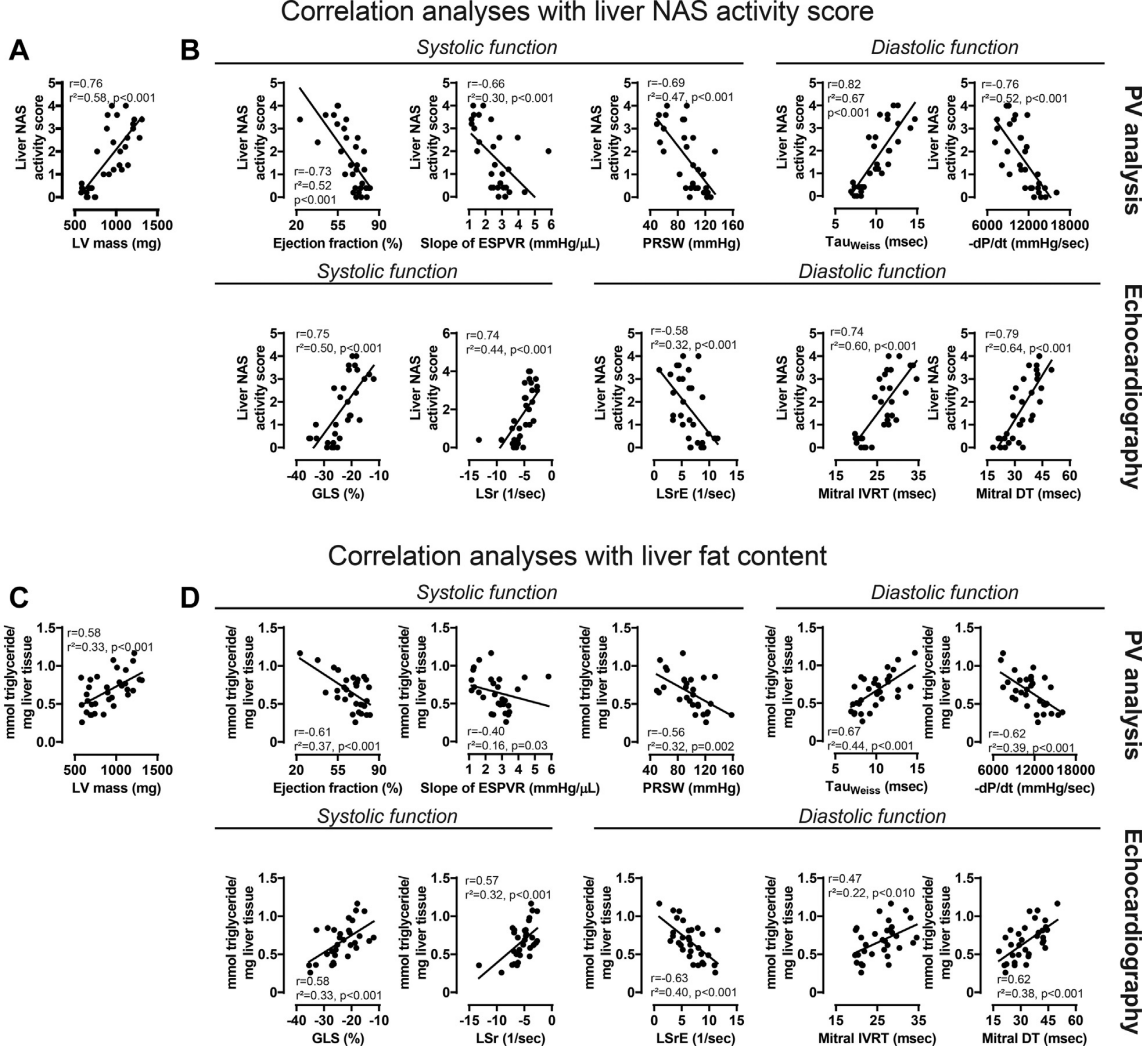
NAFLD Development Correlates With the Impairment of Cardiovascular Dysfunction Correlation analyses of liver NAS with (A) LV mass and with (B) functional parameters derived from PV analysis and echocardiography. Correlation analyses of liver triglyceride content with (C) LV mass and (D) functional parameters derived from PV analysis and echocardiography. Abbreviations as in Figure 1.

Severity of NAFLD status (NAS) positively correlated with increasing LV mass values (Figure 3A) and correlated with the impairment of different systolic (ejection fraction, slope of ESPVR, PRSW, GLS, LSr) and diastolic (Tau_Weiss_, −dP/dt, LSrE, IVRT, DT) parameters (Figure 3B). Moreover, liver triglyceride levels correlated positively with increasing LV mass values (Figure 3C) and correlated with cardiovascular systolic and diastolic dysfunction (Figure 3D).

#### Alirocumab improved age-related cardiovascular dysfunction

**Figure 4:**
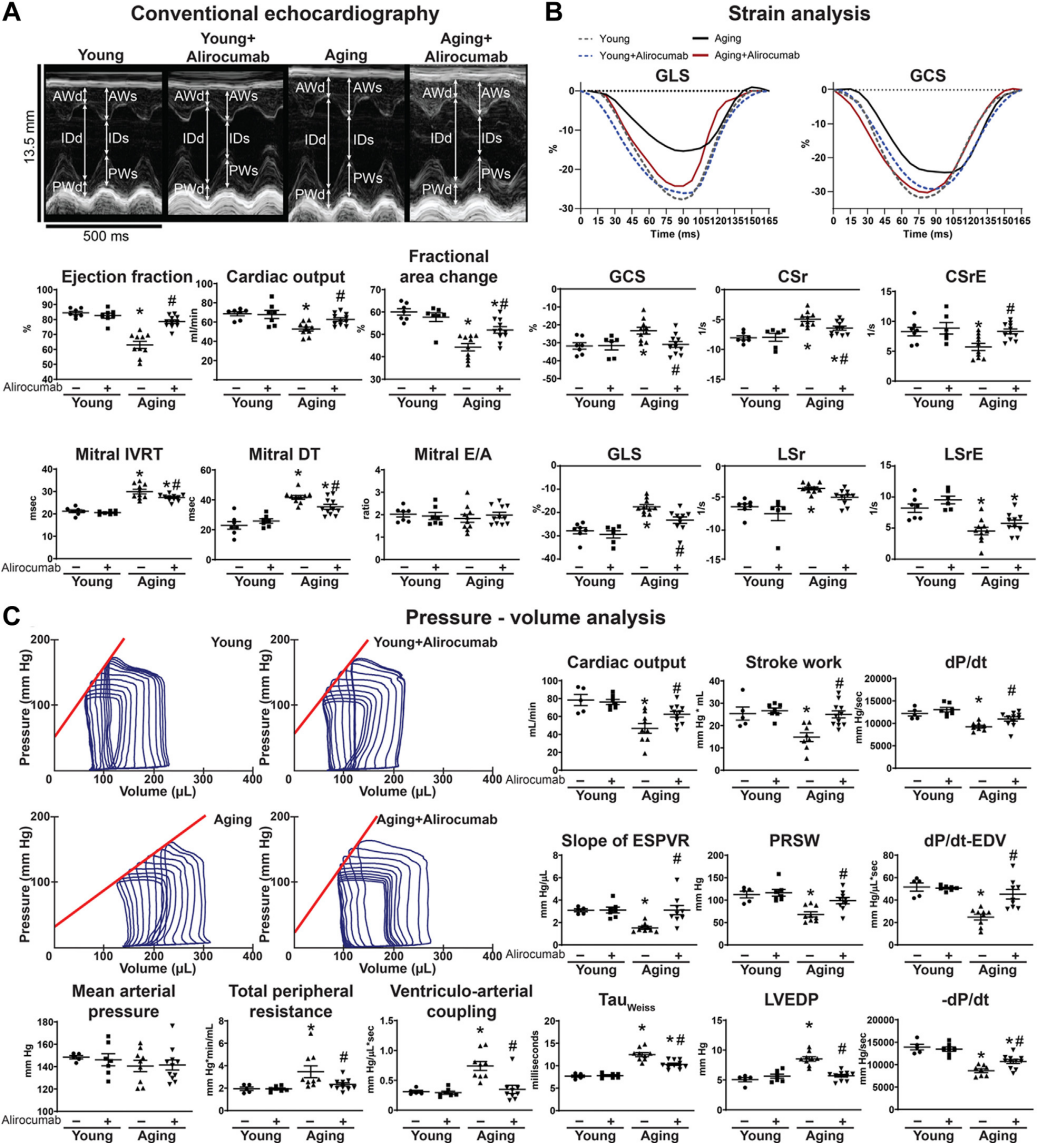
PCSK9 Inhibition Improves Age-Related Cardiovascular Dysfunction (A) Representative echocardiography recordings and echocardiography results. (B) Strain analysis. (C) PV analysis with representative recordings. ∗*P <* 0.05 vs young; #*P <* 0.05 vs aging. One-way analysis of variance with Tukey’s post hoc test. Scattered dot plots show mean ± SEM. AW = anterior wall; CSr = circumferential strain rate; CSrE = circumferential strain rate E-wave; d = diastole; dP/dt = maximal rate of left ventricular pressure rise; EDV = end-diastolic volume; E/A = E-wave to A-wave ratio; GCS = global circumferential strain; ID = internal diameter; PW = posterior wall; s = systole; other abbreviations as in Figure 1.

Conventional echocardiography showed impaired systolic (ejection fraction, cardiac output, fractional area change) and diastolic (mitral IVRT and DT) function in aging animals (Figure 4A). Speckle-tracking strain analysis supported serious impairment of contractility (global circumferential strain, circumferential strain rate, GLS, LSr) and diastolic dysfunction (circumferential early diastolic strain rate, LSrE) of the aging animals (Figure 4B). Hemodynamic investigation showed significant decrease of conventional systolic functional parameters such as cardiac output, stroke work, and maximal rate of left ventricular pressure rise (dP/dt) in the aging group (Figure 4C), while detailed analysis of pressure-volume loops revealed severe impairment of contractility (slope of ESPVR, PRSW, maximal rate of left ventricular pressure rise and end-diastolic volume relationship [dP/dt-EDV]) in the aging rats (Figure 4C). Diastolic dysfunction in aging (Tau_Weiss_, LV end-diastolic pressure, −dP/dt) was associated with unchanged mean arterial pressure values and increased peripheral resistance (Figure 4C), leading to a mismatch in ventriculoarterial coupling (Figure 4C). Hemodynamics, including systolic-diastolic function, contractility, and vascular function, were all improved by the drug treatment (Figure 4C).

#### Alirocumab improved myocardial remodeling

**Figure 5:**
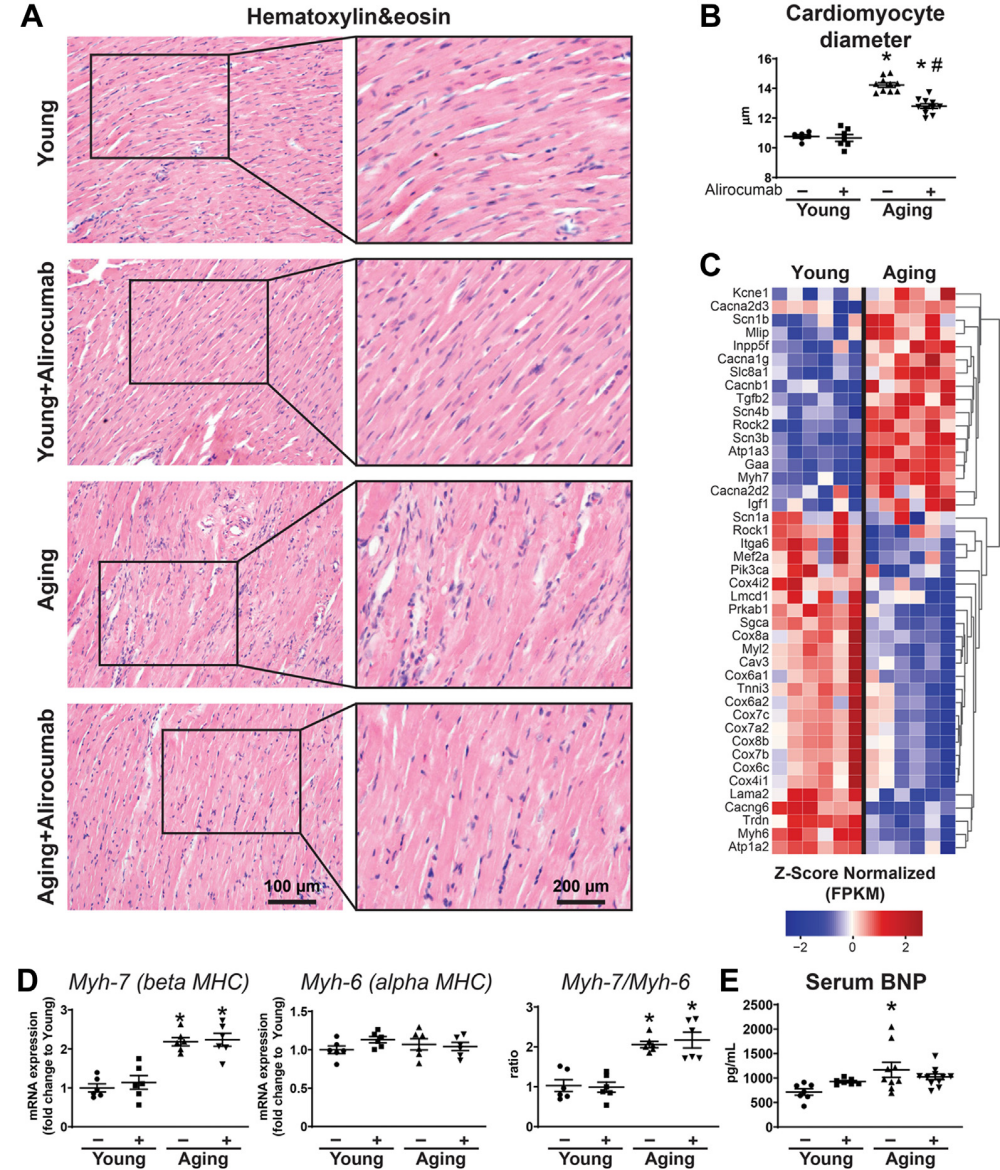
PCSK9 Inhibition Attenuates Cardiac Hypertrophy (A) Hematoxylin and eosin histology and (B) cardiomyocyte diameter. (C) Heatmap of hypertrophy- and contractility-related genes from transcriptomic analysis. (D) Gene expression levels of Myh-7 and Myh-6 (alpha and beta myosin heavy chain [MHC], respectively). (E) Serum B-type natriuretic peptide (BNP) levels. ∗*P <* 0.05 vs young; #*P <* 0.05 vs aging. One-way analysis of variance with Tukey’s post hoc test. Scattered dot plots show mean ± SEM. FPKM = fragments per kilobase of transcript per Million mapped reads; mRNA = messenger RNA; PCSK9 = proprotein convertase subtilisin/kexin type 9.

Aging was associated with cardiac hypertrophy, represented by increased cardiomyocyte diameter (Figures 5A and 5B), alteration of messenger RNA expression profile (Figure 5C), and the reactivation of the fetal gene program (Figure 5D). Higher serum B-type natriuretic peptide levels indicated the development of chronic heart failure in this group (Figure 5E). Alirocumab treatment decreased cardiac hypertrophy in aging (Figures 5A and 5B).

**Figure 6:**
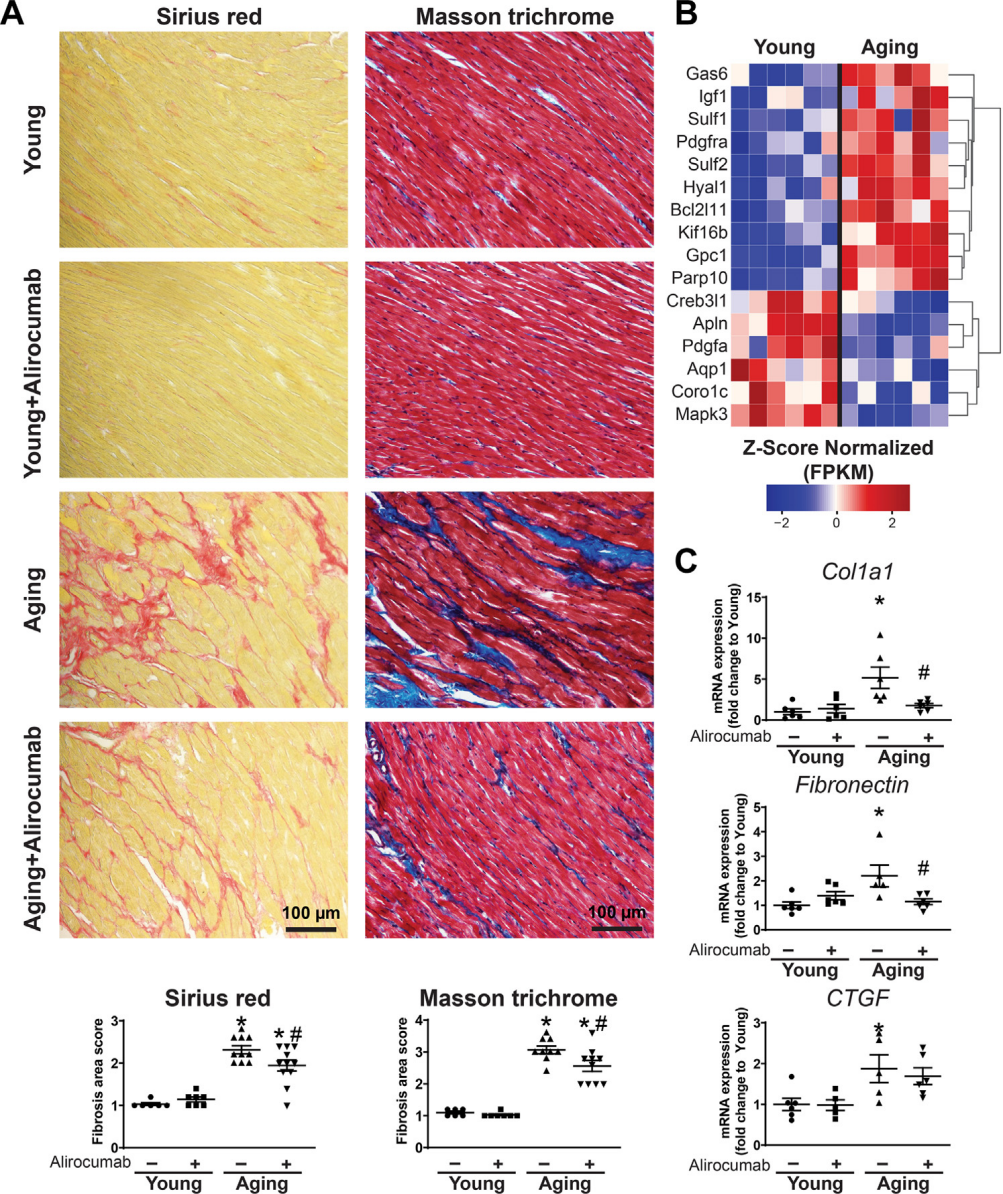
PCSK9 Inhibition Attenuates Cardiac Fibrosis (A) Sirius red and Masson trichrome histology and scoring. (B) Heatmap showing fibrosis-related genes from transcriptomic analysis. (C) Col1a1, fibronectin, and CTGF gene expression levels. ∗*P <* 0.05 vs young; #*P <* 0.05 vs aging. One-way analysis of variance with Tukey’s post hoc test. Scattered dot plots show mean ± SEM. Abbreviations as in Figures 1, 2, and 5.

On the other hand, fibrotic remodeling of the myocardium was observed in aging animals (Figure 6A). These changes were supported with a shift in messenger RNA expression profile (Figures 6B and 6C). PCSK9 inhibitor treatment prevented further progression of fibrotic remodeling in the heart (Figures 6A and 6C).

#### Alirocumab attenuated lipid peroxidation, oxidative stress, and cell death

**Figure 7:**
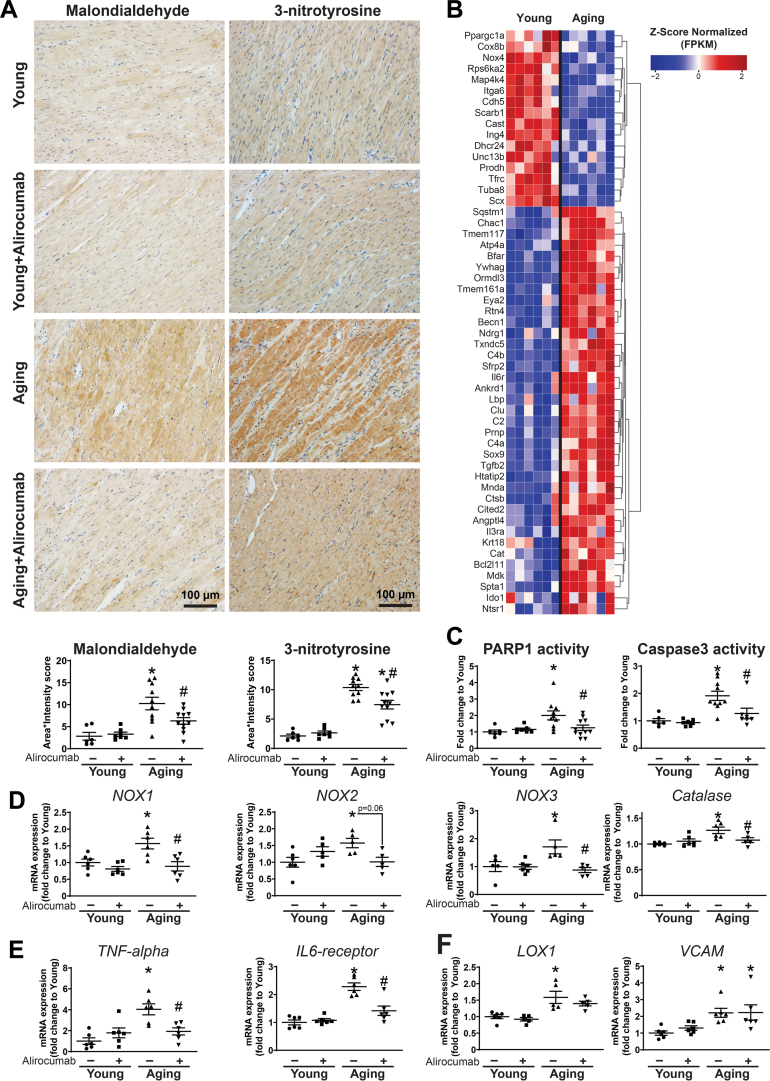
PCSK9 Inhibition Improves Age-Related Oxidative/Nitrative Stress (A) Malondialdehyde and nitrotyrosine immunohistochemistry and scoring. (B) Heatmap showing gene expression levels from transcriptomic analysis related to oxidative stress and apoptosis. (C) PARP1 and caspase3 activity levels. (D) NOX1, NOX2, NOX3 and catalase gene expression levels. (E) TNF-alpha and IL-6 receptor and (F) LOX1 and vascular cell adhesion molecule (VCAM) gene expression levels. ∗*P <* 0.05 vs young; #*P <* 0.05 vs aging. One-way analysis of variance with Tukey’s post hoc test. Scattered dot plots show mean ± SEM. Abbreviations as in Figures 1, 2, and 5.

Increasing age was associated with higher levels of oxidative (lipid peroxidation) and nitrative stress (Figure 7A) and an altered gene expression profile for genes related to oxidative stress and cell death (Figure 7B). Cell death markers PARP1 and caspase 3 showed increased activity in aging hearts (Figure 7C). Gene expression studies further supported increased oxidative stress, proinflammatory pathways, and vascular injury in the aging myocardium (Figures 7D and 7E). Alirocumab treatment effectively reduced oxidative/nitrative stress and cell death in aging hearts (Figure 7).

#### Alirocumab attenuated aging-associated mitochondrial dysfunction

**Figure 8:**
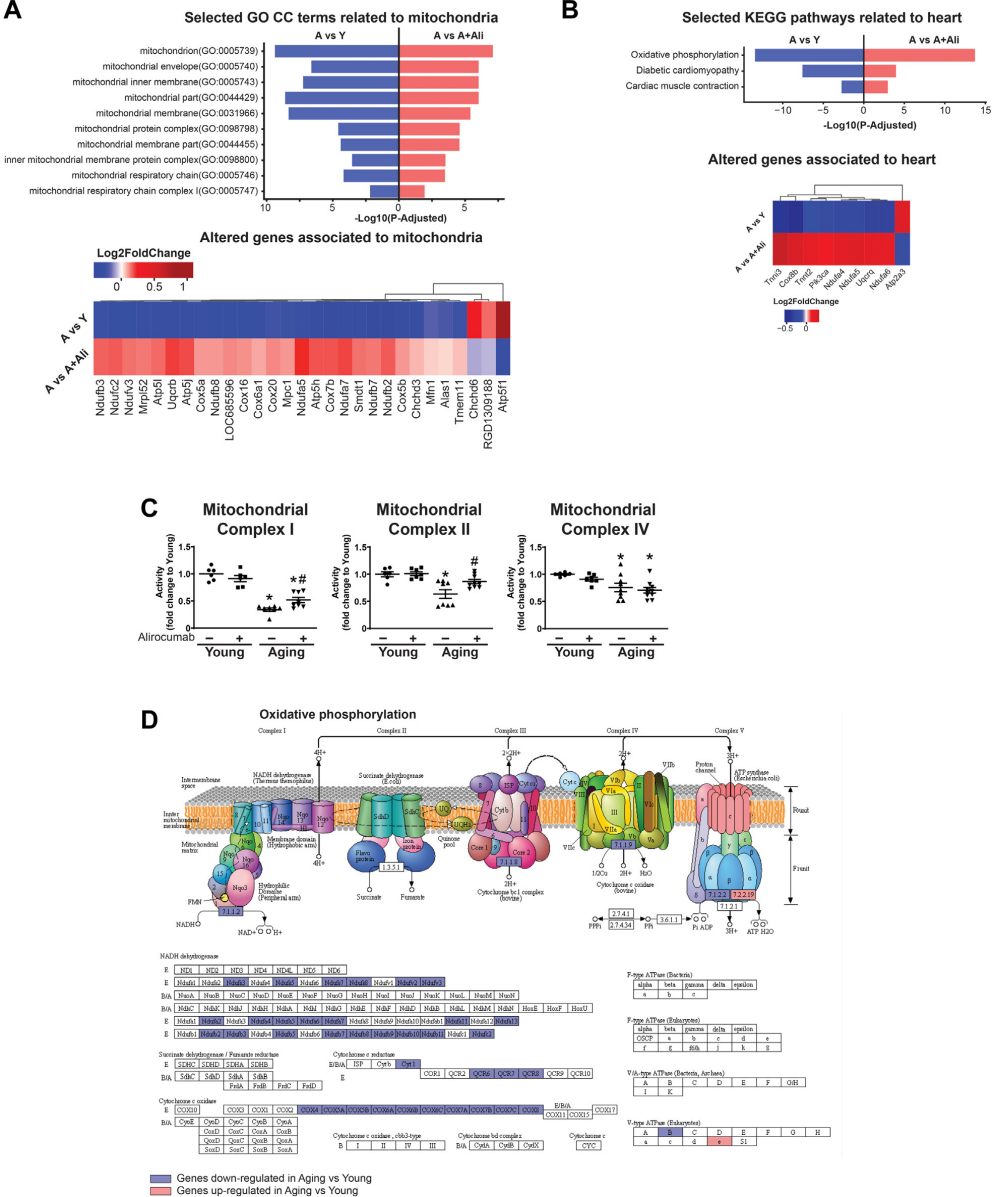
PCSK9 Inhibition Improves Mitochondrial Dysfunction (A) Selected Gene Ontology Cellular Component (GO CC) terms related to mitochondria and (B) selected Kyoto Encyclopedia of Genes and Genomes (KEGG) pathways related to heart and expression heatmap of the corresponding genes. (C) Mitochondrial complex I, II, IV activities. (D) Oxidative phosphorylation (KEGG map00190): gene expression changes in aging (A) vs young (Y). Blue = down-regulation, red = up-regulation. ∗*P <* 0.05 vs Y; #*P <* 0.05 vs A. One-way analysis of variance with Tukey’s post hoc test. Scattered dot plots show mean ± SEM. A+Ali = aging+alirocumab; PCSK9 = proprotein convertase subtilisin/kexin type 9.

The functional analysis of messenger RNA transcriptomic data revealed significant alteration and decreased expression of mitochondrion-related pathways according to the Gene Ontology Cellular Component terms (Figure 8A) and by Kyoto Encyclopedia of Genes and Genomes pathway analysis (Figures 8B and 8D). Subsequently, measurement of the activity of mitochondrial complexes showed severe mitochondrial dysfunction in the aging heart (Figure 8C). Alirocumab treatment significantly improved mitochondrial function in the aged hearts (Figure 8C).

## Discussion

Herein, we investigated the role of PCSK9 in cardiovascular dysfunction in advanced aging. We found that: 1) advanced age was associated with an increase of serum and liver PCSK9 levels; 2) blood level of PCSK9 correlated positively with the degree of cardiovascular dysfunction both in humans and experimental animals; 3) age-related fatty degeneration of liver positively correlated with serum PCSK9 levels in the rat model; 4) development of age-related NAFLD correlated with cardiovascular dysfunction; and 5) treatment with the PCSK9 inhibitor alirocumab attenuated serum lipid abnormalities, cardiac dysfunction, and cardiac and liver remodeling in aging animals, at least in part, by improving mitochondrial function and lowering oxidative/nitrative stress and inflammation.

Our study is the first to investigate plasma PCSK9 levels in a community-based screening sample, comparing young and elderly subgroups of patients not taking statins or other lipid-lowering agents. We performed a detailed characterization of systolic and diastolic cardiac function, including advanced echocardiographic parameters (ie, LV GLS and PALS). In this relatively low-risk population, we showed that elderly individuals present with a mildly decreased LV ejection fraction; however, with a pronounced LV hypertrophy and deterioration of longitudinal myocardial deformation and diastolic dysfunction. The association of these echocardiographic presentations with long-term all-cause mortality was recently established in the same community-based sample.^36^ By using iNetModels, a separate database for multiple biological networks, containing different biological, clinical, and omics data of human subjects, we identified PCSK9 as a central protein correlating positively with cholesterol levels and carotid intima-media thickness in aging, supporting the key role of PCSK9 in age-related cardiovascular and lipid abnormalities. Importantly, in our human study, blood PCSK9 levels increased with the age, a phenomenon that was confirmed in the animal cohort as well. We found an association between plasma PCSK9 levels and age-related impairment in LV diastolic function and subtle systolic dysfunction. Similarly, an age-dependent decline in cardiac contractility and active relaxation was established in a rat model of advanced aging. Increasing age proved to be one of the strongest predictors for plasma PCSK9 levels in humans, and PCSK9 level was an independent predictor for LV diastolic dysfunction. The increase in plasma PCSK9 level paralleled the cardiovascular dysfunction in humans and in the preclinical model. The latter phenomenon was confirmed by echocardiographic analyses (ie, longitudinal and circumferential strain and strain rate parameters) and a detailed pressure-volume analysis. The observed phenotypic presentation of cardiac aging shares the most frequent echocardiographic features accompanying heart failure with preserved ejection fraction. Although the elderly subgroup of this population is not a heart failure cohort, investigating the link between the phenotype and the overt disease is of high clinical interest.

Others reported the liver as the major site of PCSK9 production with several orders of magnitude more production than in any other organ.^37^ Comparable to these data, our animal studies showed abundant levels of PCSK9 in the liver, while it was further increased during aging, leading to lower expression of LDLR and dysregulation of cholesterol metabolism in the animals. Our data suggested that expression levels of PCSK9 in the cardiac tissue were very low in human bulk, single-nuclei, and spatial transcriptomic data as well as in mouse and rat tissues. Consistently with the important role of PCSK9 production in the liver, Food and Drug Administration–approved inclisiran, which targets PCSK9 synthesis in the liver, reduces LDL cholesterol levels to similar extent as the monoclonal antibodies targeting PCSK9 in humans (by approximately 50%).^38^

The relationship between elevated PCSK9 levels and hepatic fat accumulation and cardiovascular health is of significant research interest. In recent years, NAFLD and nonalcoholic steatohepatitis development were shown to be associated with cardiovascular dysfunction and remodeling.^39^, ^40^, ^41^, ^42^ Increased PCSK9 levels were observed to be associated with hepatic fat accumulation in humans.^20^ Moreover, disarrangement of LDLR expression is associated with hepatic steatosis^37^ and LDLR is thought to be a major regulator of liver steatosis.^43^ On the other hand, gain-of-function mutations of PCSK9 result in familial hypercholesterolemia and increased cardiovascular risk, while loss-of-function mutations are associated with better lipid profile and confer to lower cardiovascular risk.^44^ Importantly, PCSK9 inhibition was shown to protect against alcoholic^30^ and nonalcoholic liver steatosis.^43^ However, little is known about the role of PCSK9 in age-related steatohepatitis. In our current animal study, we observed increased levels of PCSK9 and the development of NAFLD features of the liver including fat accumulation, inflammation, and oxidative stress. Importantly, fat accumulation in the liver showed positive correlation with serum PCSK9 levels. In concert with the previous observations, degeneration of the liver (represented by NAS and fat content) correlated with the impairment of cardiovascular function in rats.

Recently, PCSK9 inhibitors were shown to have added benefit in the treatment of cardiovascular diseases. Monoclonal antibodies targeted against the circulating plasma PCSK9 lowered cardiovascular risk on top of conventional lipid-lowering therapies.^22^^,^^24^ Although modulating LDL cholesterol levels is the major site of effect of PCSK9,^21^ additional direct or indirect effects may include a proinflammatory effect on white blood cells^14^ or macrophages,^45^ regulation of vascular endothelial and smooth muscle cell proliferation,^27^ increasing the expression of oxidized LDLRs in endothelium^27^ or macrophages,^46^ mitochondrial DNA damage,^47^ and inflammation.^46^ Activation of the oxidized LDLR resulted in the induction of PCSK9 expression and decreased function in cardiomyocytes.^48^ Our animal study confirmed the dysregulation of cholesterol metabolism in aging, while it was paralleled with increased oxidized LDL levels, a major contributor to cardiomyocyte and vascular stress.^49^ Alongside these, myocardial lipid peroxidation, oxidative stress, and cell death markers were up in our aging group, probably leading to altered mitochondrial metabolism and subsequently impaired mitochondrial function and cellular senescence with characteristic changes of the myocardial transcriptome.

PCSK9 inhibitor alirocumab exerted anti-inflammatory effects in the aging liver, which is in line with a recent report in which silencing PCSK9 repressed oxidized LDLR expression and inflammatory cell activation.^50^ Moreover, it prevented hepatic LDLR degradation and effectively improved cholesterol levels in aging animals, thereby possibly removing the most important precursor molecule for oxidized LDL production, without causing liver injury or having adverse effect on aging-associated pathophysiological processes. Most importantly, age-related cardiac dysfunction was effectively attenuated by the drug, most likely by attenuation of the myocardial lipid peroxidation, oxidative stress, and mitochondrial dysfunction and consequent remodeling (hypertrophy and fibrosis) of the heart. A possible explanation for the beneficial cellular and cardiac effects may also involve a better hepatic status including less inflammation, halted fatty degeneration, and overall better cholesterol and oxidative status. These in turn will lead to better myocardial mitochondrial function, improved myocardial oxidative state, and reduced progression of cardiac remodeling.

The very low (close to zero) local PCSK9 expression in cardiac tissues (both in young and aging) and high hepatic expression, which is further increased with age, suggest that the liver is the primary source for age-related increased serum PCSK9 levels and is possibly the primary site of action of PCSK9 inhibitors. However, direct cardiovascular effects of the drug cannot be ruled out either.

### Study limitations

The human study was a community-based screening sample not designed specifically for the investigation of the relationship between blood PCSK9 levels, age, and cardiac function.

Although multiple analyses showed negligible levels of PCSK9 in young and aging hearts, enzyme-linked immunosorbent assay showed very low but detectable levels of PCSK9 protein in aging rat hearts compared with the young hearts. A possible explanation is that, despite our best efforts to perfuse the hearts, remaining blood in heart tissue samples could have caused the measured higher levels in the aging rat cardiac samples. Moreover, measured protein levels in hearts were at the lower end of the detection range of the method used; thus, values could have been overestimated. Although the aging liver showed increased PCSK9 protein expression and a characteristic aging phenotype of the liver tissue with steatohepatitis, we did not investigate whether elevated PCSK9 contributed to the development of NASH-like features or the other way around, namely PCSK9 levels were increased due to inflammation and oxidative stress in the hepatic tissue. Of note, aging-associated increased oxidative stress or inflammation could have had promoted PCSK9 synthesis in the aging livers, however this hypothesis has not been tested in the current study. On the other hand, cardiac fibroblast activity, senescence-associated beta-galactosidase activity in isolated cardiomyocytes or hepatocytes, the role of the renin-angiotensin system, or in vivo cell death or mitochondrial function, or effects of PCSK9 overexpression during aging were not explored. Importantly, reducing oxidative stress, inflammation, and the levels of cholesterol alongside with lower ox-LDL levels in the circulation is a reasonable explanation for the (indirect) beneficial effects of PCSK9 inhibition on the heart. However, direct cardiac and hepatic effects cannot be ruled out.

## Conclusions

**Figure 9:**
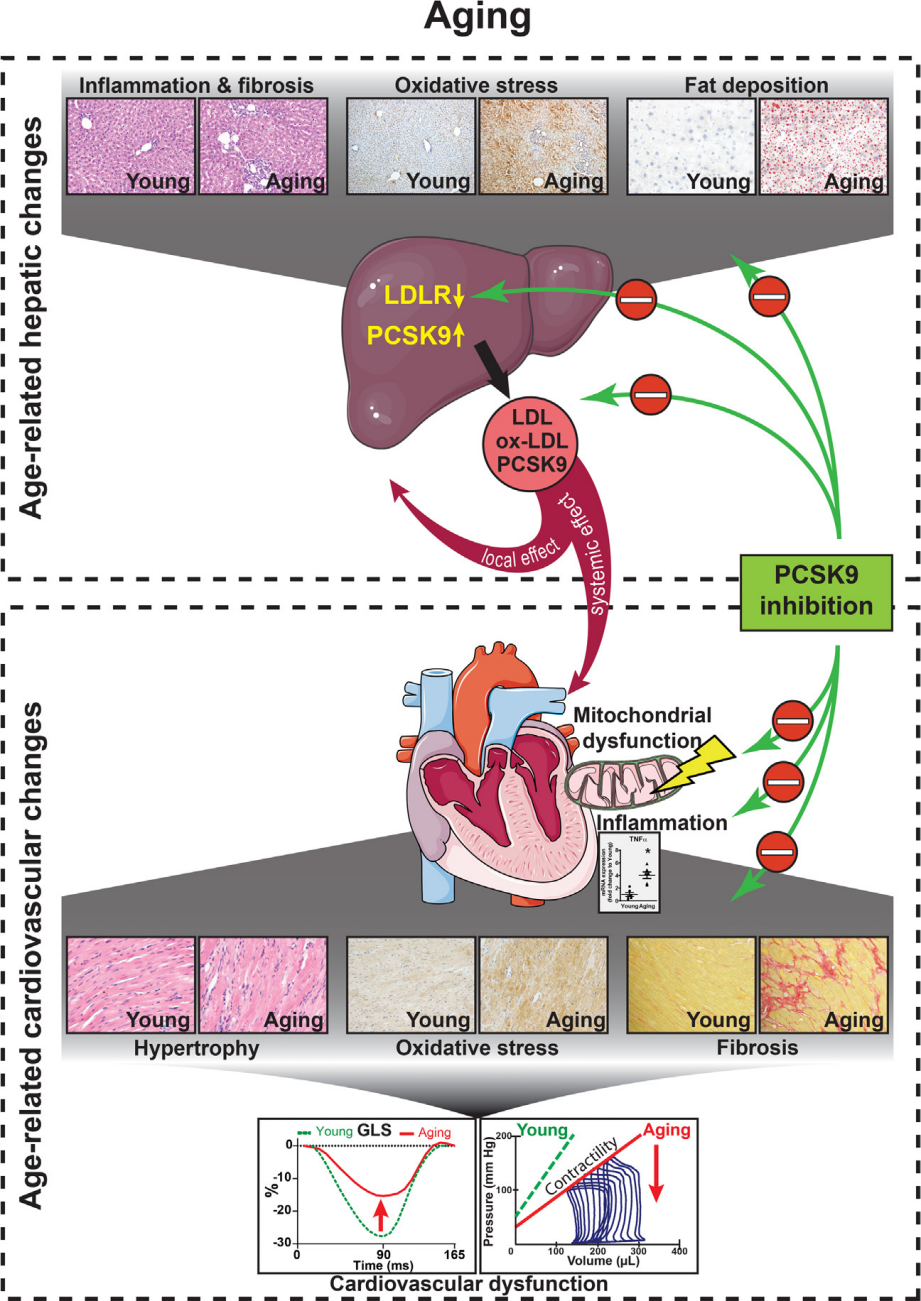
Proposed Mechanism of Beneficial Effects of PCSK9 Inhibition on Aging-Related Cardiovascular Dysfunction Aging is associated with characteristic changes of the hepatic tissue, such as inflammation, fibrosis, oxidative stress, and fat deposition. These pathological events induce increased PCSK9 production and consequently decreased number of LDLRs. LDL, oxidized low-density lipoprotein (ox-LDL), and PCSK9 are secreted from the liver and promote local and systemic effects, possibly contributing to the development of age-related cardiovascular changes including mitochondrial injury and dysfunction, myocardial hypertrophy, oxidative stress, inflammation (TNF-α) and fibrotic remodeling, which eventually culminate in cardiovascular dysfunction. PCSK9 inhibition, as a novel interventional strategy, might improve age-related cardiac and hepatic pathologies by acting on systemic and local targets. Student’s unpaired *t* test. Scattered dot plots show mean ± SEM, ∗*P <* 0.05. Abbreviations as in Figures 1 and 2.

In summary, age-associated development of NAFLD is associated with elevated PCSK9 levels reflecting a mechanistic link for the observed development of left ventricular dysfunction. Age proved to be one of the strongest predictors of plasma PCSK9 levels, while increased blood PCSK9 level was an independent predictor for the development of LV diastolic dysfunction in elderly individuals. Animal data supported the human observations, whereas elevated PCSK9 levels correlated with LV dysfunction and a characteristic aging cardiac phenotype, which was attenuated by PCSK9 inhibition (Figure 9). Upon that, PCSK9 might serve not only as a drug target but also as a potential biomarker of age-related cardiac dysfunction if confirmed by larger scale future studies.

Perspectives **COMPETENCY IN MEDICAL KNOWLEDGE:** Cardiovascular diseases are among the leading causes of mortality and morbidity in the elderly population. With the continuous increase of age, the prevalence of cardiovascular diseases is expected to further grow in the coming decades. Oxidative stress, inflammation, mitochondrial dysfunction, and myocardial remodeling are hallmarks of age-related cardiovascular functional decline.

**TRANSLATIONAL OUTLOOK:** Although conventional lipid-lowering therapies (including statins) have greatly decreased cardiovascular mortality and morbidity, they remained the leading cause of death in older individuals. Development of fatty liver and blood PCSK9 levels correlate with the functional decline of the cardiovascular system in aging, suggesting that its inhibition can improve cardiovascular function. PCSK9 inhibitors may exert beneficial effects beyond cardiovascular effects (attenuation of myocardial oxidative stress, mitochondrial dysfunction, and remodeling) on extracardiac tissues such as the liver (decreased fat deposition, inflammation, and oxidative stress).

## Funding Support and Author Disclosures

The study was supported by the intramural program of National Institute on Alcohol Abuse and Alcoholism/National Institutes of Health Grant 1ZIAAA000375-17 (to Dr Pachar) and partly by the National Institute on Alcohol Abuse and Alcoholism/National Institutes of Health Grant R01 AG072895 (to WXD). The research was supported by project NKFIH-1277-2/2020 by the Thematic Excellence Programme (2020-4.1.1.-TKP2020) of the Ministry for Innovation and Technology in Hungary, within the framework of the Bioimaging Thematic Programme of Semmelweis University. Project no. RRF-2.3.1-21-2022-00003 has been implemented with the support provided by the European Union. Drs Fabian and Kovacs have received personal fees from Argus Cognitive, Inc, outside the submitted work. Dr Tokodi was formerly an employee of Argus Cognitive, Inc. Dr Merkely has received grants from Boston Scientific and Medtronic and personal fees from Biotronik, Abbott, AstraZeneca, Novartis, and Boehringer Ingelheim, outside the submitted work. All other authors have reported that they have no relationships relevant to the contents of this paper to disclose.
