# Supplementary material for: PCSK9, A Promising Novel Target for Age-Related Cardiovascular Dysfunction
Source: JACC Basic Transl Sci. 2023 Sep 13;8(10):1334–53. doi: 10.1016/j.jacbts.2023.06.005 (PMC10715889; doi:10.1016/j.jacbts.2023.06.005)

**Supplemental material to:**

**Title:** PCSK9, a promising novel target for age-related cardiovascular dysfunction

**Brief title:** PCSK9 in aging heart

**Authors:** Csaba Matyas MD PhD^1,9^*, Eszter Trojnar MD PhD^1^*, Suxian Zhao MD PhD^1^, Muhammad Arif PhD^1^, Partha Mukhopadhyay PhD^1^, Attila Kovacs MD PhD^2^, Alexandra Fabian MD^2^, Marton Tokodi MD PhD^2^, Zsolt Bagyura MD PhD^2^, Bela Merkely MD PhD^2^, Laszlo Kohidai MD PhD^3^, Eszter Lajko PharmD PhD^3^, Angela Takacs PharmD^3^, Yong He PharmD PhD ^4^, Bin Gao MD PhD^4^, Janos Paloczi PhD^1^, Falk W. Lohoff MD^5^, György Haskó MD PhD^6^, Wen-Xing Ding PhD^7,8^, Pal Pacher MD PhD^1^

^1^Laboratory of Cardiovascular Physiology and Tissue Injury, National Institute on Alcohol Abuse and Alcoholism, National Institutes of Health, Bethesda, Maryland, USA

^2^Heart and Vascular Center, Semmelweis University, Budapest, Hungary

^3^Department of Genetics, Cell and Immunobiology, Semmelweis University, Budapest, Hungary

^4^Laboratory of Liver Diseases, National Institute on Alcohol Abuse and Alcoholism, National Institutes of Health, Bethesda, Maryland, USA

^5^Section on Clinical Genomics and Experimental Therapeutics, National Institute on Alcohol Abuse and Alcoholism, National Institutes of Health, Bethesda, Maryland, USA

^6^Department of Anesthesiology, Columbia University, New York, New York, USA

^7^Department of Pharmacology, Toxicology and Therapeutics, University of Kansas Medical Center, Kansas City, Kansas, USA

^8^Department of Internal Medicine, University of Kansas Medical Center, Kansas City, Kansas, USA

^9^Department of Medical Imaging, Medical School, University of Pecs, Pecs, Hungary

*Equal contribution

**Detailed Supplemental methods:**

**Human study**

**Patient population**

The Budakalász Study is a voluntary, longitudinal, population-based screening program in the Central Hungarian region to collect information on the citizens' health status and cardiovascular risk profile and discover new potential cardiovascular risk factors(1). The study was approved by the Hungarian Scientific and Research Ethics Committee of the Medical Research Council (Approval No: 8224-0/2011/EKU [265/PI/11]). Procedures followed the ethical standards of the responsible committee on human experimentation (institutional and national) and the Helsinki Declaration of 1975 (5th revision). Written informed consent was obtained from all study participants. Its protocol consists of several health-related questionnaires, non-invasive tests (anthropometry, echocardiography, carotid duplex scan, blood pressure measurement, ankle-brachial index, and others), venous blood sample collection, and laboratory tests. Between 2011 and 2014, 2420 subjects (30% of Budakalász’s population, male: 41.2%, average age 54.8 years) participated in the program. In the current study, we aimed to define two age categories: a young group with subjects below 35 years of age; and an elderly group above 65 years of age. Exclusion criteria were: (1) subjects taking statins or any other lipid-lowering agents; (2) no serum samples available; (3) no echocardiographic recordings available; and (4) no apical 4-chamber view recordings available appropriate for speckle-tracking analysis based on the criteria below (Figure 1A).

**Echocardiography**

Transthoracic echocardiographic examinations were performed using a commercially available ultrasound system (Vivid i, 3Sc probe, GE Vingmed Ultrasound, Horten, Norway). A standard acquisition protocol consisting of 2D loops from parasternal, apical, and subxiphoid views was applied. All analyses were performed offline at the Echocardiography Core Laboratory of the Semmelweis University by experienced, certified readers. Left ventricular (LV) internal diameters and wall thicknesses; left atrial (LA) end-systolic volume (by the Simpson's method); mitral inflow velocities such as early (E) and late diastolic (A) peak velocities, their ratio, and E wave deceleration time; systolic (s′) and early diastolic (e′) velocities of the mitral lateral and septal annulus (by tissue Doppler imaging); average E/e′; right ventricular (RV) basal diameter, tricuspid annular plane systolic excursion (TAPSE), peak velocity of the tricuspid regurgitation jet, right atrial (RA) end-systolic volume (by the Simpson's method); and peak aortic outflow velocity were measured according to current guidelines(2).LV mass was calculated using the Devereux formula and was indexed to body surface area. LV end-diastolic and end-systolic volumes were measured by Simpson's method on apical 4-chamber view acquisitions, and ejection fraction (EF) was calculated accordingly.

**Speckle-tracking echocardiography**

Beyond conventional echocardiographic measurements, the current analysis pipeline necessitated high-quality, ECG-gated images appropriate for two-dimensional speckle-tracking analysis. The targeted frame rate was above 50 FPS, and adequate focus position, depth, sector size, and time-gain compensation settings should be applied. Recordings were exported to a standalone workstation for offline analysis with commercially available software (2D Cardiac Performance Analysis v1.2, TomTec Imaging Systems, Unterschleissheim, Germany). The speckle-tracking analysis was performed by a single experienced operator blinded to patient characteristics. After manual delineation of the LV endocardial surface on apical 4-chamber view recordings, the software tracked the region of interest throughout three consecutive cardiac cycles. If the operator detected low endocardial tracking fidelity visually, the contour was realigned, and the calculation was repeated to a maximum of three attempts. Subjects with poor tracking quality were excluded as determined visually based on the tracking pattern or the lack of explicit end-systolic peak value of the time-strain curve. Peak systolic longitudinal strains of the 6 LV segments averaged over three cardiac cycles were used to calculate the corresponding global longitudinal strain (GLS) value. Similarly, the LA endocardial surface was manually contoured on apical 4-chamber view and tracked by the same dedicated software package (2D Cardiac Performance Analysis v1.2) to calculate peak atrial longitudinal strain (PALS) representing the reservoir function of the LA. Lower (more negative) longitudinal strain values represent more deformation (better function).

**The determination of plasma PCSK9 levels**

Blood samples were drawn in ethylenediaminetetraacetic acid (EDTA) treated tubes and then subjected to centrifugation. Plasma samples were preserved at -80°C until the analysis of PCSK9 using a Quantikine ELISA kit (R&D Systems, MN, USA, #SPC900). The level of PCSK9 was measured according to the manufacturer’s instructions. Prior to the measurement, the plasma samples were diluted (1:20) as suggested. Optical density was read at 450 and 540 nm and then readings at 540 nm were subtracted from readings at 450 nm to correct the data. All experiments were performed in duplicates.

**Network analysis of PCSK9 and aging from iNetModels**

PCSK9 and aging multi-omics network was retrieved from a separate database of multi-omics data called the iNetModels(3) (https://inetmodels.com), specifically overall longitudinal data from SCAPIS-SciLifeLab Wellness Profiling Study(4). This dataset includes 101 subjects (males and females) with age ranging from 50-65 years old.

**Human Transcriptomics Data**

Human RNA-seq data were obtained from The Genotype-Tissue Expression (GTEx) Project Portal on 02/08/2022 and dbGaP accession number phs000424.v8.p2 on 02/08/2022. TPM (transcript per-kilobase million) data were filtered to include the selected tissues from subjects with no history of diseases or cause of death associated with the tissues. The excluded conditions were:

1. Heart: cardiac arrest, myocardial infarction, coronary syndrome, cardiac asystole, cardiopulmonary failure, congestive heart failure, multi-organ failure, and other ischemic and idiopathic cardiovascular diseases.
2. Liver: Hepatitis, diabetes (type 1 and type II), liver abscess, failure, fatty liver syndrome, inherited liver insufficiency, acute/chronic hepatic insufficiency, necrobacillosis, rupture, end-stage liver disease (ESLD)

Moreover, we excluded samples from patients with history of hypertension and body mass index > 30. The sample IDs of the selected samples and their corresponding tissues are included in Suppl. Table 4 (for organ type comparison) and 5 (for age group comparison). The TPM values of each sample were normalized by the mean TPM of the heart tissue to get the fold changes.

The Single-Nuclei transcriptomics data was taken from the Heart Cell Atlas (https://heartcellatlas.org)(5) and spatial transcriptomics was from 10X Genomics left ventricular heart data (https://support.10xgenomics.com/spatial-gene-expression/datasets/1.0.0/V1_Human_Heart). Both of them were re-analyzed using Scanpy 1.9.3 in Python 3.7 using the same parameters as described by the Heart Cell Atlas.

**Animal study**

**Treatment protocol**

Male F344/DuCrl (Fisher) rats were obtained from the National Institute on Aging. The study was reviewed and approved by the Institutional Animal Care and Use Committee of the National Institute on Alcohol Abuse and Alcoholism, conformed to the National Institutes of Health guidelines on animal experimenting (“Guide for the Care and Use of Laboratory Animals” prepared by the National Academy of Sciences and published by the National Institutes of Health (NIH publication 86–23 revised 1985). Rats were kept in a specific pathogen-free facility, with constant temperature (22±2°C), humidity and 12-12 hours alternating light cycle. They received humane care, regular chow diet and were non-fasted at the time of experimentation. Experiments were carried out during daylight conditions. Young (3 months old) and Aging (19 months old) animals were divided into four groups and treated with either the vehicle (physiological saline) or alirocumab (diluted in physiological saline solution, 50mg/kg/week s.c., Regeneron purchased via DVR Pharmacy, NIH) for 6 weeks: Young (vehicle-treatment, n=7), Young+Alirocumab (alirocumab-treatment, n=7), Aging (vehicle-treatment, n=10), Aging+Alirocumab (alirocumab-treatment, n=11). At the end of the 6-week study period functional measurements and tissue collection were carried out (Figure 1.). Serum samples and snap frozen tissue (liver, heart) samples were stored at -80°C for biochemical measurements, and formalin-fixed samples (embedded in paraffin, stored at room temperature (RT)) were prepared for histological purposes(6). Additionally, liver tissue was collected in O.C.T compound (Sakura Finetek, Torrance, CA, USA) on dry ice for frozen sectioning.

**Echocardiography and speckle tracking myocardial strain analysis**

Echocardiography was done on anesthetized (nosecone, isoflurane 1-2%) rats held on a heating pad to maintain body temperature at 37+0.5°C in a supine position with the use of a Visualsonics Vevo3100 ultrasound machine (Fujifilm Visualsonics, Toronto, Canada) coupled with the MX201 transducer(7,8). Parasternal long- and short axis images were recorded (2D and M-mode), mitral flow was measured and the following parameters were calculated: heart rate (HR), left ventricular (LV) anterior and posterior wall thickness in systole (s) and diastole (d)(LVAWs, LVAWd, LVPWs, LVPWd), LV internal diameter in ‘s’ and ‘d’ (LVIDs, LVIDd) , end-diastolic, end-systolic and stroke volumes, LV mass, ejection fraction (EF), fractional area change, cardiac output (CO), mitral isovolumic relaxation time (IVRT), deceleration time (DT), early (E) to atrial (A) velocity ratio (E/A ratio).

Echocardiographic images were acquired at a constant frame rate (~130-150 frames per second). Three consecutive cardiac cycles were analyzed in a blinded fashion with Vevolab 3.2.6 and VevoStrain softwares (Fujifilm Visualsonics, Toronto, Canada). Strain analysis was done as earlier(7-9). Global circumferential strain (GCS) and circumferential systolic strain rate (CSr) were analyzed on short-axis views of the mid-papillary level, while global longitudinal strain (GLS) and longitudinal systolic strain rate (LSr) were assessed on parasternal long axis view images. Circumferential and longitudinal early diastolic strain rate (CSrE and LSrE, respectively) were manually analyzed on the strain rate curves. After manual delineation of the endocardial border, the analysis software automatically divided the region of interest into six segments with the Time-to-Peak analysis feature of the software. These segments were tracked during the analysis. In case of poor tracking adherence, manual correction was applied and again analyzed by the software. Strain curves were investigated without the application of temporal smoothing filters. Global strain, strain rate values were obtained by averaging the data across all six segments. Asynchronicity between the segments was not detected in any of the animals throughout the analysis.

**Invasive hemodynamics and pressure-volume analysis**

Measurements were done under isoflurane anesthesia (1-2%, intubated, mechanically ventilated)(10,11). Mean arterial pressure (MAP), CO, stroke work (SW), total peripheral resistance (TPR), maximal slope of systolic pressure increment (dP/dt) and decrement (-dP/dt), time constant of LV pressure decay (Tau_Weiss_), LV end-diastolic pressure (LVEDP) were measured. The slope of the LV end-systolic PV relationship (ESPVR; quadratic model), the preload recruitable stroke work (PRSW), and the dP/dt_max_-end-diastolic volume (dP/dt-EDV) were calculated as load- and HR-independent indices of cardiac contractility. Ventriculo-arterical coupling (VAC) was calculated as a ratio of arterial elastance and end-systolic elastance (slope of ESPVR quadratic model).

**Measurement of serum liver injury markers, cholesterol, oxLDL and BNP**

Serum aspartate and alanine transaminase (AST, ALT) and cholesterol levels were determined by the chemistry services of the Department of Laboratory Medicine, NIH. OxLDL levels were determined in equal amounts of 1:20 diluted serum samples by the use of a commercially available kit (LSBio, Seattle, WA, USA, Rat OxLDL ELISA Kit, #LS-F25192) according to the manufacturer’s guidelines. Serum BNP was measured in 1:4 diluted serum samples according to the manufacturer’s instructions (BNP ELISA kit, #RAB0386, SigmaAldrich).

**Determination of serum, liver and heart PCSK9 levels**

PCSK9 levels were measured by a commercially available kit (MBL Intl., Woburn, MA, USA, Circulex Mouse/Rat PCSK9 ELISA Kit, #CY-8078). Serum samples were diluted 1:50, and equal amounts were measured according to the manufacturer’s recommendations.

Frozen liver and heart samples were homogenized in Tissue Protein Extraction Reagent (ThermoFisher, Waltham, MA, USA), protein concentration was determined by BCA method (Pierce BCA Protein Assay Kit, ThermoFisher) and equal amounts of protein were loaded on a microplate, and the above kit was used according to the instructions.

**Liver triglyceride assay**

Liver samples were homogenized and liver triglyceride levels were measured according to the manufacturer’s instructions (Triglyceride Quantification Kit, #K622, Biovision, Milpitas, CA, USA).

**Western blotting**

Snap frozen LV or liver tissue were used for western blotting experiments. The samples were homogenized using RIPA buffer (ThermoFisher) containing phosphatase and protease inhibitors (Phosstop; Complete proteinase inhibitor (Roche, Basel, Switzerland), protein concentration was determined by BCA assay. Lysates were mixed in Laemmli (BioRad, Hercules, CA, USA) buffer supplemented with beta-mercaoptoethanol (Sigma Aldrich, Burlington, MA, USA), heated to 95°C for 5min (liver LDL receptor) or 40°C for 30min (heart mitochondrial complexes), and equal protein amounts were loaded on the gel (Criterion TGX 4-15% or 4-20%, BioRad). Proteins were transferred to nitrocellulose membrane by Trans-Blot Turbo Transfer System (BioRad). Transfer efficiency was checked by Pierce™ Reversible Protein Stain Kit (ThermoFisher). Membranes were blocked in SuperBlock (TBS) buffer (ThermoFisher) for 1h at RT. Then, membranes were subjected to overnight incubation with the primary antibody at 4°C (liver LDL receptor: 1/1000, #AB180623, Abcam; mitochondrial complexes: 1/2000, #AB110413, Abcam; GAPDH for housekeeping, appropriately with or without stripping: 1/10000, #MAB374, SigmaAldrich or 1/1000, #2118, CST). Chemiluminescent imaging was done on G:BOX Mini platform (Syngene, Frederick, MD, USA) with the use of SuperSignal Pico and Dura Kits (ThermoFisher) combined. Images were analyzed using GeneTools (Syngene). Results were normalized to GAPDH and expressed as fold change of the Young group.

**Myocardial PARP1, caspase 3 and mitochondrial complex activities**

Equal amount of protein from heart lysates were used. PARP1 (PARP1 Enzyme Activity Assay, #17-10149, Millipore, Burlington, MA, USA), caspase 3 (Caspase-3 Assay Kit, #AB39383, Abcam) and mitochondrial complex I, II, IV activities (Complex activity assay kits, #AB109721, #AB109908, #AB109911, Abcam) were assessed according to the manufacturer’s instructions. Results were expressed as fold change to the Young group.

**RNA isolation and quantitative real-time PCR**

Left ventricular and liver samples were homogenized as described previously(12) by using the Direct-zol RNA Miniprep Kit including on-column DNase treatment (Zymo Research, Irvine, CA, USA). RNA was reverse transcribed to cDNA using the High-Capacity cDNA Reverse Transcription Kit (Thermo Fisher Scientific). Quantitative real-time PCR experiments were performed with SyberSelect PCR Master Mix (Thermo Fisher Scientific) on an ABI 7900HT Real-time PCR Instrument (Applied Biosystems, Foster City, CA, USA). Reactions were performed in duplicates. Specifity of transcript amplification was confirmed by dissociation curve profiles. Relative quantification of the target gene expression was calculated with the comparative CT method (2^−ΔΔCt^)(6). Target gene expression levels were compared to the expression of a group of housekeeping genes including beta-actin, beta-2-microglobulin, ribosomal protein lateral stalk subunit P0 and hypoxanthine phosphoribosyltransferase 1. Primers used to detect target gene expression levels are listed in Suppl. Table 6.

**mRNA sequencing**

Raw data (raw reads) of FASTQ format were firstly processed through fastp(13). In this step, clean data (clean reads) were obtained by removing reads containing adapter and poly-N sequences and reads with low quality from raw data. At the same time, Q20, Q30 and GC content of the clean data were calculated. All the downstream analyses were based on the clean data with high quality. Reference genome and gene model annotation files were downloaded from genome website browser (NCBI/UCSC/Ensembl) directly. Paired-end clean reads were mapped to the reference genome using HISAT2 software. HISAT2(14) uses a large set of small GFM indexes that collectively cover the whole genome. These small indexes (called local indexes), combined with several alignment strategies, enable rapid and accurate alignment of sequencing reads. Because transcriptome annotations are still incomplete, most RNA-seq studies will reveal novel genes and transcripts.

The Stringtie(15) was used to assemble the set of transcript isoforms of each bam file obtained in the mapping step. Gffcompare can compare Stringtie assemblies to reference annotation files and help sort out new genes from known ones. Featurecounts was used to count the read numbers mapped of each gene, including known and novel genes. And then Fragments Per Kilobase of transcript per Million mapped reads (FPKM) of each gene was calculated based on the length of the gene and reads count mapped to this gene. FPKM considers the effect of sequencing depth and gene length for the reads count at the same time, and is currently the most commonly used method for estimating gene expression levels.

To perform differential expression analysis for the transcriptomic data, the raw count files from HISAT2 were used as an input to DESeq2(20) package in R, and genes with P-Value < 0.05 were considered as significantly differentially expressed genes. The gene-level statistics (Log2 Fold Changes and P-Value) from DESeq2 were then used to perform functional analysis, together with the gene-set collection from KEGG(21) and Gene Ontology (GO)(22) downloaded from Enrichr(23,24) library. The functional analyses were performed using PIANO(25) package in R. Benjamin-Hochberg False Discovery Rate (FDR) was used for the adjustment. KEGG or GO terms with FDR<0.05 were considered significant. Visualization of the results was done using Seaborn(26) package in Python 3.7.

To further validate PCSK9 gene expression (TPM) in mouse heart and liver (GSE153485) a separate whole-transcriptomics database from an already published, independent study was used(16).

**Histology**

Standard procedures were used to deparrafinate 5 µm thin liver and heart paraffin-embedded sections. For all immuno-histochemistries (IHC), antigen retrieval was performed in 1x citrate buffer (#6414208; Electron Microscopy Sciences, Hatfield, PA, USA) at 95ºC for 15 minutes in a pressure cooker. All IHCs were evaluated according to an Area*Intensity scoring system (Area: 1- 0-25%, 2- 25-50%, 3- 50-75%, 4- 75-100%; Intensity: 1- nothing, 2- weak, 3- mild, 4- strong) on 5 areas under 200x magnification(10). Average values of area*intensity scores were used for statistics.

Steatohepatitis and liver fibrosis (NAFLD activity score [NAS]) was graded on hematoxylin&eosin (H&E)-stained liver sections as previously described (Scores: Steatosis: 0: <5%, 1: 6-33%, 2: 34-66%, 3: >66%; Activity grade: Inflammation: 0: none, 1: 1-2foci/20x field, 2: 2-4 foci/20 field, 3: >4 foci/20x field, Ballooning: 0: none, 1: rare/few, 2: many; Fibrosis: 0: none, 1: central or portal, 2: central and portal, 3: bridging fibrosis, 4: cirrhosis)(17). IHC for 4-hydroxy-2-nonenal (4-HNE) was done according to the manufacturer’s instructions (Vector M.O.M kit ImmPRESS Peroxidase Polymer; #MP-2400, Vector Laboratories, Burlingame, CA, USA). Sections were incubated in BloxAll (Vector) to block endogenous peroxidase activity. Primary antibody was applied overnight at 4ºC in 1/200 dilution. DAB (Vector) was used for staining development.

Oil red O was performed to assess the liver fat content(18) on 10 µm thin frozen sections.

H&E-stained cardiac sections were used to assess cardiomyocyte diameter as described earlier(10). Sirius red and Masson trichrome stainings were used to evaluate cardiac fibrosis with the following scoring system: positive area: 1- 0-25%, 2- 25-50%, 3- 50-75%, 4- 75-100%. Average of 5 areas under 200x magnification was used for statistical purposes. IHC for Malondialdehyde (MDA) was performed according to the manufacturer’s instructions (Vector M.O.M kit). Sections were blocked in BloxAll (Vector) and primary antibody was applied overnight at 4ºC in 1/100 dilution. 3-nitrotyrosine (3-NT) IHC was performed according to the manual (Vector ImmPRESS Excel Staining Kit; Peroxidase, Anti-Rabbit IgG; #MP-7601, Vector). After BloxAll incubation, primary antibody was applied overnight at 4ºC in 1/500 dilution. DAB (Vector) was used for staining development.

**Acknowledgement**

The Genotype-Tissue Expression (GTEx) Project was supported by the Common Fund of the Office of the Director of the National Institutes of Health, and by NCI, NHGRI, NHLBI, NIDA, NIMH, and NINDS.

**References:**

1. Bagyura Z, Kiss L, Edes E et al. [Cardiovascular screening programme in the Central Hungarian region. The Budakalasz Study]. Orv Hetil 2014;155:1344-52.

2. Lang RM, Badano LP, Mor-Avi V et al. Recommendations for cardiac chamber quantification by echocardiography in adults: an update from the American Society of Echocardiography and the European Association of Cardiovascular Imaging. J Am Soc Echocardiogr 2015;28:1-39 e14.

3. Arif M, Zhang C, Li X et al. iNetModels 2.0: an interactive visualization and database of multi-omics data. Nucleic Acids Res 2021;49:W271-W276.

4. Tebani A, Gummesson A, Zhong W et al. Integration of molecular profiles in a longitudinal wellness profiling cohort. Nat Commun 2020;11:4487.

5. Litvinukova M, Talavera-Lopez C, Maatz H et al. Cells of the adult human heart. Nature 2020;588:466-472.

6. Matyas C, Erdelyi K, Trojnar E et al. Interplay of Liver-Heart Inflammatory Axis and Cannabinoid 2 Receptor Signaling in an Experimental Model of Hepatic Cardiomyopathy. Hepatology 2020;71:1391-1407.

7. Kovacs A, Olah A, Lux A et al. Strain and strain rate by speckle-tracking echocardiography correlate with pressure-volume loop-derived contractility indices in a rat model of athlete's heart. Am J Physiol Heart Circ Physiol 2015;308:H743-8.

8. Matyas C, Kovacs A, Nemeth BT et al. Comparison of speckle-tracking echocardiography with invasive hemodynamics for the detection of characteristic cardiac dysfunction in type-1 and type-2 diabetic rat models. Cardiovasc Diabetol 2018;17:13.

9. Bauer M, Cheng S, Jain M et al. Echocardiographic speckle-tracking based strain imaging for rapid cardiovascular phenotyping in mice. Circ Res 2011;108:908-16.

10. Matyas C, Nemeth BT, Olah A et al. Prevention of the development of heart failure with preserved ejection fraction by the phosphodiesterase-5A inhibitor vardenafil in rats with type 2 diabetes. Eur J Heart Fail 2017;19:326-336.

11. Pacher P, Nagayama T, Mukhopadhyay P, Batkai S, Kass DA. Measurement of cardiac function using pressure-volume conductance catheter technique in mice and rats. Nat Protoc 2008;3:1422-34.

12. Trojnar E, Erdelyi K, Matyas C et al. Cannabinoid-2 receptor activation ameliorates hepatorenal syndrome. Free Radic Biol Med 2020;152:540-550.

13. Chen S, Zhou Y, Chen Y, Gu J. fastp: an ultra-fast all-in-one FASTQ preprocessor. Bioinformatics 2018;34:i884-i890.

14. Kim D, Paggi JM, Park C, Bennett C, Salzberg SL. Graph-based genome alignment and genotyping with HISAT2 and HISAT-genotype. Nat Biotechnol 2019;37:907-915.

15. Pertea M, Pertea GM, Antonescu CM, Chang TC, Mendell JT, Salzberg SL. StringTie enables improved reconstruction of a transcriptome from RNA-seq reads. Nat Biotechnol 2015;33:290-5.

16. Arif M, Klevstig M, Benfeitas R et al. Integrative transcriptomic analysis of tissue-specific metabolic crosstalk after myocardial infarction. Elife 2021;10.

17. Chalasani N, Younossi Z, Lavine JE et al. The diagnosis and management of nonalcoholic fatty liver disease: Practice guidance from the American Association for the Study of Liver Diseases. Hepatology 2018;67:328-357.

18. He Y, Rodrigues RM, Wang X et al. Neutrophil-to-hepatocyte communication via LDLR-dependent miR-223-enriched extracellular vesicle transfer ameliorates nonalcoholic steatohepatitis. J Clin Invest 2021;131.

**Suppl. Table 1.** Human study data: list of medications

| Medication | Non-missing (n) | Young | Elderly | p-value |
| --- | --- | --- | --- | --- |
| **Beta blockers, n (%)** | 209 | 1 (1.0) | 24 (22.0) | 6.47E-07 |
| **ACE-I or ARB, n (%)** | 209 | 3 (3.0) | 45 (41.3) | 3.54E-12 |
| **Loop diuretics, n (%)** | 209 | 0 (0.0) | 2 (1.8) | 0.49 |
| **Any diuretics, n (%)** | 209 | 0 (0.0) | 13 (11.9) | <0.001 |
| **Aspirin, n (%)** | 209 | 0 (0.0) | 11 (10.1) | <0.001 |
| **Amiodarone, n (%)** | 209 | 0 (0.0) | 1 (0.9) | 1 |
| **Other anti-arrhythmic drugs, n (%)** | 209 | 0 (0.0) | 6 (5.5) | 0.029 |
| **Vitamin K antagonists or novel oral anticoagulants, n (%)** | 209 | 0 (0.0) | 17 (15.6) | 9.84E-06 |

Abbreviations: Angiotensin-converting-enzyme inhibitors (ACE-I), Angiotensin receptor blockers (ARB). Variables are presented as n (%).

**Suppl. Table 2.** Gene and protein expression levels of PCSK9

| **Species** | **Method** | **Heart** | **Liver** |
| --- | --- | --- | --- |
| Rat (F344/DuCrl, Fisher, young) | ELISA | 0.0078±0.0038 (n=6)  (pg/mg protein) | 0.3807±0.0488 (n=6)  (pg/mg protein) |
| Rat (F344/DuCrl , Fisher, young) | Whole-transcriptomics | 0.000±0.00 (FPKM) (n=6) | 23.034±1.87 (FPKM) (n=6) |
| Mouse (C57Bl/6N) young | Whole-transcriptomics | 0.02 ± 0.01 (TPM) (n= 10) | 10.22 ± 2.07 (TPM) (n=10) |
| Human (GTEx) | RNA-seq | 0.022 ± 0.00 (TPM) (n=97) | 25.58 ± 2.31 (TPM) (n=64) |

Table lists protein levels of PCSK9 (proprotein convertase subtilisin/kexin type 9) from young and rat hearts and livers used in our rat study, and FPKM (fragments per kilobase million) data of *Pcsk9* from young and aging rat heart and liver (GSE210371) whole-transcriptomics data from the matched animals used in this study. Moreover, table presents TPM (transcript per-kilobase million) data of PCSK9 of the human heart (left ventricle) and liver from subjects included in GTEx database and TPM data of *Pcsk9* from mouse heart and liver (GSE153485) whole-transcriptomics data from the matched animals from an independent study. ELISA: enzyme linked immunosorbent assay. Values are mean±SEM.

**Suppl. Table 3.** Cardiac expression levels of PCSK9 in Young and Aging groups

| **Human transcriptomic data** | Young | Aging |
| --- | --- | --- |
| ALL (GTEX) | 0.0189 ± 0.004 (TPM) (n=45) | 0.020 ± 0.003 (TPM) (n=149) |
| Age (years) | below 40 | above 60 |
|  |  |  |
| Healthy (GTEX) | 0.0168 ± 0.004 (TPM) (n=32) | 0.0215 ± 0.005 (TPM) (n=58) |
| Age (years) | below 40 | above 60 |
|  |  |  |
| **Rat transcriptomic data** | Young | Aging |
| Rat (F344/DuCrl , Fisher,) | 0.000 ± 0.00 (FPKM) (n=6) | 0.006 ± 0.01 (FPKM) (n=6) |
| **Rat ELISA** | Young | Aging |
| Rat (F344/DuCrl , Fisher, | 0.0078±0.0038  (pg/mg protein) (n=6) | 0.0460±0.0076  (pg/mg protein) (n=10) |

Table lists gene expression levels of PCSK9 (proprotein convertase subtilisin/kexin type 9) from young and aging humans or rats. Values are mean±SEM.

**Suppl. Table 4.** GTEx sample IDs used for Suppl. Table 2.

| **Sample ID** | **Tissue** | **Sample ID** | **Tissue** |
| --- | --- | --- | --- |
| GTEX-U3ZH-0326-SM-3DB7A | Heart | GTEX-1J8EW-0926-SM-A9G33 | Heart |
| GTEX-1F75I-1126-SM-9WYUO | Heart | GTEX-YB5K-0126-SM-5IFJ2 | Heart |
| GTEX-OIZI-0926-SM-E6CHF | Heart | GTEX-17F9E-0926-SM-7IGMG | Heart |
| GTEX-OIZF-0326-SM-3MJGY | Heart | GTEX-1OKEX-0726-SM-DPRYE | Heart |
| GTEX-QVJO-1926-SM-2S1QZ | Heart | GTEX-O5YT-0326-SM-32PKA | Heart |
| GTEX-PLZ5-0626-SM-2I5F8 | Heart | GTEX-1IKOE-1026-SM-ARZMF | Heart |
| GTEX-NPJ8-0426-SM-2HMK6 | Heart | GTEX-ZQUD-1526-SM-7IGO2 | Heart |
| GTEX-QLQ7-0526-SM-2I5G3 | Heart | GTEX-1PPH7-2026-SM-E9J3N | Heart |
| GTEX-17KNJ-0826-SM-793A4 | Heart | GTEX-11DXX-0326-SM-5PNWC | Heart |
| GTEX-1F6I4-0726-SM-7PC2P | Heart | GTEX-14DAQ-1126-SM-664MT | Heart |
| GTEX-145LV-0326-SM-5Q5BW | Heart | GTEX-RWSA-0626-SM-2XCBD | Heart |
| GTEX-1LGOU-0726-SM-E9TIY | Heart | GTEX-WRHU-1226-SM-4E3IJ | Heart |
| GTEX-1LH75-0426-SM-E9TJF | Heart | GTEX-13VXT-1126-SM-5LU3A | Heart |
| GTEX-WFG8-0626-SM-3GILJ | Heart | GTEX-1GN1W-0326-SM-9JGHF | Heart |
| GTEX-11ZVC-0426-SM-5CVLD | Heart | GTEX-11I78-0826-SM-5A5K4 | Heart |
| GTEX-131XH-0226-SM-5LZVB | Heart | GTEX-139YR-0826-SM-5LZXY | Heart |
| GTEX-13NZB-0226-SM-5KM1T | Heart | GTEX-15SHU-1126-SM-7KUMB | Heart |
| GTEX-13FTW-1026-SM-5L3E3 | Heart | GTEX-YFC4-0526-SM-5ZZVI | Heart |
| GTEX-13W3W-0426-SM-5SI9D | Heart | GTEX-T2IS-0426-SM-32QPE | Heart |
| GTEX-14PN3-0526-SM-69LQF | Heart | GTEX-PWCY-0526-SM-5P9HG | Heart |
| GTEX-1HBPH-0126-SM-CNNQ7 | Heart | GTEX-1CB4E-0526-SM-7MXTK | Heart |
| GTEX-RNOR-0826-SM-2TF5C | Heart | GTEX-1IDJF-0726-SM-CJI1U | Heart |
| GTEX-WHPG-0826-SM-3NMBF | Heart | GTEX-WFG7-0726-SM-3GIKO | Heart |
| GTEX-13OW5-1126-SM-5J1NR | Heart | GTEX-14PHX-2126-SM-6EU2U | Heart |
| GTEX-QEG5-0926-SM-2TC64 | Heart | GTEX-13FHP-1026-SM-5K7Y2 | Heart |
| GTEX-1JJ6O-2126-SM-CNNP6 | Heart | GTEX-1IDJU-0726-SM-CNPQ9 | Heart |
| GTEX-1GMR3-0326-SM-7P8QT | Heart | GTEX-1F48J-1026-SM-7P8SO | Heart |
| GTEX-WEY5-0426-SM-3GIKT | Heart | GTEX-REY6-1026-SM-2TF4Y | Heart |
| GTEX-132NY-1726-SM-5EGKK | Heart | GTEX-XPT6-0126-SM-4B65S | Heart |
| GTEX-11EMC-0726-SM-5EGJO | Heart | GTEX-WHSE-0926-SM-3NMBS | Heart |
| GTEX-1CAMS-1326-SM-79OJR | Heart | GTEX-14PJM-1126-SM-6LLHK | Heart |
| GTEX-111FC-0826-SM-5GZWO | Heart | GTEX-1IDJE-0326-SM-CM2TD | Heart |
| GTEX-POMQ-0326-SM-2I5FO | Heart | GTEX-1211K-0626-SM-5FQUZ | Heart |
| GTEX-U4B1-0326-SM-3DB8K | Heart | GTEX-QESD-0526-SM-2I5G5 | Heart |
| GTEX-132AR-2026-SM-5IJG4 | Heart | GTEX-QMRM-0526-SM-2I5GA | Heart |
| GTEX-1GN1V-0826-SM-9KNU6 | Heart | GTEX-14A5I-1226-SM-5NQBW | Heart |
| GTEX-O5YW-0326-SM-2I5EI | Heart | GTEX-PSDG-0926-SM-2I5FP | Heart |
| GTEX-11EM3-0626-SM-5H12Z | Heart | GTEX-ZDTS-1326-SM-6LLHE | Heart |
| GTEX-1GF9U-0426-SM-7RHHA | Heart | GTEX-RN64-0826-SM-2TC62 | Heart |
| GTEX-WHWD-0426-SM-3LK83 | Heart | GTEX-11TT1-1326-SM-5PNYM | Heart |
| GTEX-13RTJ-0726-SM-5QGQN | Heart | GTEX-1KANC-0426-SM-CXZK5 | Heart |
| GTEX-U8XE-1126-SM-3DB8W | Heart | GTEX-UPK5-0326-SM-CYPR4 | Heart |
| GTEX-13U4I-0826-SM-5SIBD | Heart | GTEX-1LGRB-1126-SM-CNPP2 | Heart |
| GTEX-XXEK-0926-SM-4BRWH | Heart | GTEX-1H11D-1226-SM-9WPQ2 | Heart |
| GTEX-R55E-1026-SM-2TC5S | Heart | GTEX-SNMC-0126-SM-2XCFO | Heart |
| GTEX-12WSK-0626-SM-5LZUJ | Heart | GTEX-P44G-0826-SM-2I5ES | Heart |
| GTEX-P4QS-0326-SM-2I3EU | Heart | GTEX-18D9U-1126-SM-72D5Y | Heart |
| GTEX-1C2JI-0526-SM-7EWEQ | Heart | GTEX-1K9T9-1126-SM-D3L9E | Heart |
| GTEX-QEG4-0426-SM-33HC3 | Heart | GTEX-1BAJH-1526-SM-7RHGI | Liver |
| GTEX-UPK5-1426-SM-4JBHH | Liver | GTEX-RNOR-1426-SM-48FDJ | Liver |
| GTEX-ZTPG-1426-SM-51MT3 | Liver | GTEX-XXEK-1126-SM-4BRUX | Liver |
| GTEX-11TT1-1726-SM-5EQLJ | Liver | GTEX-18A66-2026-SM-7189C | Liver |
| GTEX-139YR-0226-SM-5IFEM | Liver | GTEX-1J8EW-1626-SM-C1YQ8 | Liver |
| GTEX-YB5E-0326-SM-5IFHU | Liver | GTEX-T6MN-1226-SM-3NMA5 | Liver |
| GTEX-15SHU-1826-SM-7KUKS | Liver | GTEX-REY6-1226-SM-48FDR | Liver |
| GTEX-SJXC-1226-SM-4DM78 | Liver | GTEX-1LH75-0926-SM-EAZ5C | Liver |
| GTEX-1GN1U-0926-SM-9WPPY | Liver | GTEX-13PVR-0126-SM-5S2PY | Liver |
| GTEX-13NZB-0626-SM-5IFH6 | Liver | GTEX-Q734-0326-SM-48U15 | Liver |
| GTEX-XBEC-1526-SM-4AT68 | Liver | GTEX-R53T-0326-SM-48FEC | Liver |
| GTEX-Y5LM-0426-SM-4VBRO | Liver | GTEX-YFC4-1526-SM-5IFJS | Liver |
| GTEX-13112-1426-SM-5EGH8 | Liver | GTEX-1GN73-1126-SM-9OSW5 | Liver |
| GTEX-Y5V5-0926-SM-4VBPZ | Liver | GTEX-1K9T9-0726-SM-CXZJZ | Liver |
| GTEX-1H11D-0826-SM-9OSWB | Liver | GTEX-1JJEA-1526-SM-CNNPK | Liver |
| GTEX-12696-0826-SM-5EGGE | Liver | GTEX-14PJO-1726-SM-68719 | Liver |
| GTEX-1QP66-0226-SM-DPRXS | Liver | GTEX-QESD-2026-SM-447BI | Liver |
| GTEX-OIZF-0826-SM-3MJGO | Liver | GTEX-18A7A-1526-SM-72D69 | Liver |
| GTEX-145LU-1326-SM-5LU9N | Liver | GTEX-1JJ6O-0826-SM-CXZJL | Liver |
| GTEX-131YS-1626-SM-5HL6C | Liver | GTEX-U8XE-1526-SM-4E3HT | Liver |
| GTEX-14JG1-1626-SM-664NH | Liver | GTEX-131XH-0626-SM-5LZWH | Liver |
| GTEX-144GM-1326-SM-5LU5E | Liver | GTEX-13N2G-0926-SM-5IFGJ | Liver |
| GTEX-132NY-0926-SM-5P9G3 | Liver | GTEX-12KS4-1326-SM-5LUB3 | Liver |
| GTEX-1HBPH-2626-SM-A96TV | Liver | GTEX-O5YT-0826-SM-3TW8N | Liver |
| GTEX-RWSA-1426-SM-47JXA | Liver | GTEX-13FTW-1126-SM-5J2NV | Liver |
| GTEX-ZF2S-3026-SM-4WWCH | Liver | GTEX-P44G-1126-SM-3NM9D | Liver |
| GTEX-14PHX-0526-SM-664NW | Liver | GTEX-1NV8Z-1626-SM-DPRYT | Liver |
| GTEX-QV44-0326-SM-C1YQX | Liver | GTEX-132AR-0426-SM-5IFH8 | Liver |
| GTEX-P78B-1326-SM-3P611 | Liver | GTEX-11ZVC-0726-SM-5FQT9 | Liver |
| GTEX-11NUK-1226-SM-5P9GM | Liver | GTEX-14DAQ-1726-SM-5S2R2 | Liver |
| GTEX-QEG4-1826-SM-CKZN9 | Liver | GTEX-1CB4F-1726-SM-7MGXH | Liver |
| GTEX-13113-1326-SM-5GCOI | Liver | GTEX-13VXU-0926-SM-5IFFH | Liver |
| GTEX-12WSM-0726-SM-5GCOW | Liver |  |  |

**Suppl. Table 5.** GTEx sample IDs used for Suppl. Table 3.

| SAMPLE ID | Tissue type | Age group (years) |
| --- | --- | --- |
| GTEX-U3ZH-0326-SM-3DB7A | Heart | below40 |
| GTEX-OIZF-0326-SM-3MJGY | Heart | above60 |
| GTEX-QVJO-1926-SM-2S1QZ | Heart | above60 |
| GTEX-1RB15-0326-SM-E6CQB | Heart | above60 |
| GTEX-OOBJ-0326-SM-33HBO | Heart | above60 |
| GTEX-1HBPM-1226-SM-A9SM8 | Heart | above60 |
| GTEX-15EOM-5007-SM-793DG | Heart | below40 |
| GTEX-1JN6P-1126-SM-CKZON | Heart | above60 |
| GTEX-12BJ1-0226-SM-5LUA2 | Heart | above60 |
| GTEX-QLQ7-0526-SM-2I5G3 | Heart | above60 |
| GTEX-148VJ-1526-SM-5Q5DU | Heart | above60 |
| GTEX-PX3G-0326-SM-2I3EO | Heart | below40 |
| GTEX-16YQH-0926-SM-793BC | Heart | below40 |
| GTEX-1LGOU-0726-SM-E9TIY | Heart | below40 |
| GTEX-WFG8-0626-SM-3GILJ | Heart | below40 |
| GTEX-O5YV-0326-SM-2I5H2 | Heart | above60 |
| GTEX-13FTZ-0226-SM-5K7X6 | Heart | above60 |
| GTEX-WQUQ-1426-SM-3MJFD | Heart | above60 |
| GTEX-13NZB-0226-SM-5KM1T | Heart | below40 |
| GTEX-1ICG6-0626-SM-ACKWS | Heart | above60 |
| GTEX-13W3W-0426-SM-5SI9D | Heart | above60 |
| GTEX-1AX9J-1026-SM-72D6E | Heart | above60 |
| GTEX-14PN3-0526-SM-69LQF | Heart | above60 |
| GTEX-1122O-0826-SM-5GICV | Heart | above60 |
| GTEX-13O1R-1226-SM-5J1NU | Heart | above60 |
| GTEX-13OW5-1126-SM-5J1NR | Heart | above60 |
| GTEX-QEG5-0926-SM-2TC64 | Heart | below40 |
| GTEX-1JJ6O-2126-SM-CNNP6 | Heart | above60 |
| GTEX-11EMC-0726-SM-5EGJO | Heart | above60 |
| GTEX-1A3MX-1326-SM-72D58 | Heart | above60 |
| GTEX-1MCC2-0326-SM-EV7AK | Heart | below40 |
| GTEX-14PJ6-0726-SM-69LOL | Heart | above60 |
| GTEX-1OKEX-0726-SM-DPRYE | Heart | above60 |
| GTEX-O5YT-0326-SM-32PKA | Heart | below40 |
| GTEX-1IKOE-1026-SM-ARZMF | Heart | below40 |
| GTEX-ZQUD-1526-SM-7IGO2 | Heart | below40 |
| GTEX-11DXX-0326-SM-5PNWC | Heart | above60 |
| GTEX-14DAQ-1126-SM-664MT | Heart | above60 |
| GTEX-Y5V6-0826-SM-4VBRU | Heart | above60 |
| GTEX-13O3O-1526-SM-5KM1C | Heart | above60 |
| GTEX-ZPCL-0426-SM-DNZZ4 | Heart | above60 |
| GTEX-13VXT-1126-SM-5LU3A | Heart | below40 |
| GTEX-14C5O-1326-SM-5S2UW | Heart | above60 |
| GTEX-147JS-1526-SM-5ZZWF | Heart | above60 |
| GTEX-1399R-1926-SM-5K7X8 | Heart | below40 |
| GTEX-1212Z-0626-SM-5FQTB | Heart | above60 |
| GTEX-1POEN-0626-SM-EVR4B | Heart | below40 |
| GTEX-11TUW-1026-SM-5GU7D | Heart | above60 |
| GTEX-T2IS-0426-SM-32QPE | Heart | below40 |
| GTEX-PWCY-0526-SM-5P9HG | Heart | below40 |
| GTEX-1CB4E-0526-SM-7MXTK | Heart | above60 |
| GTEX-1IDJF-0726-SM-CJI1U | Heart | below40 |
| GTEX-WFG7-0726-SM-3GIKO | Heart | below40 |
| GTEX-1IDJU-0726-SM-CNPQ9 | Heart | above60 |
| GTEX-ZDTT-0726-SM-4WKFK | Heart | above60 |
| GTEX-REY6-1026-SM-2TF4Y | Heart | above60 |
| GTEX-1J8QM-0426-SM-AHZ3X | Heart | above60 |
| GTEX-1JN1M-1026-SM-CNPOS | Heart | above60 |
| GTEX-XPT6-0126-SM-4B65S | Heart | below40 |
| GTEX-WHSE-0926-SM-3NMBS | Heart | below40 |
| GTEX-111FC-0826-SM-5GZWO | Heart | above60 |
| GTEX-POMQ-0326-SM-2I5FO | Heart | below40 |
| GTEX-1GN1V-0826-SM-9KNU6 | Heart | above60 |
| GTEX-RTLS-0826-SM-2TF5Q | Heart | above60 |
| GTEX-OHPL-0326-SM-33HC8 | Heart | above60 |
| GTEX-11EM3-0626-SM-5H12Z | Heart | below40 |
| GTEX-1MUQO-1226-SM-E9TJK | Heart | above60 |
| GTEX-1EH9U-0826-SM-7IGPT | Heart | above60 |
| GTEX-U8XE-1126-SM-3DB8W | Heart | below40 |
| GTEX-1269C-0826-SM-5N9EM | Heart | above60 |
| GTEX-R55E-1026-SM-2TC5S | Heart | below40 |
| GTEX-N7MT-1326-SM-2I3FV | Heart | above60 |
| GTEX-1QCLZ-0926-SM-E6CQZ | Heart | above60 |
| GTEX-P4QS-0326-SM-2I3EU | Heart | above60 |
| GTEX-1H3NZ-1126-SM-9WYT9 | Heart | above60 |
| GTEX-14A6H-0226-SM-5Q5DX | Heart | below40 |
| GTEX-13X6K-1826-SM-5O9CR | Heart | above60 |
| GTEX-SNMC-0126-SM-2XCFO | Heart | below40 |
| GTEX-P44G-0826-SM-2I5ES | Heart | below40 |
| GTEX-1N2EF-2826-SM-E6CQF | Heart | above60 |
| GTEX-QEG4-0426-SM-33HC3 | Heart | below40 |
| GTEX-13OVG-0526-SM-5K7YD | Heart | above60 |
| GTEX-ZDTS-1326-SM-6LLHE | Heart | above60 |
| GTEX-13N1W-1026-SM-5IJC5 | Heart | above60 |
| GTEX-11TT1-1326-SM-5PNYM | Heart | below40 |
| GTEX-1LC47-1026-SM-D3LAH | Heart | above60 |
| GTEX-17F9Y-1326-SM-7MGW9 | Heart | above60 |
| GTEX-1314G-0126-SM-5LZUL | Heart | below40 |
| GTEX-1211K-0626-SM-5FQUZ | Heart | above60 |
| GTEX-QESD-0526-SM-2I5G5 | Heart | below40 |
| GTEX-13O3O-1826-SM-5IFGW | Liver | above60 |
| GTEX-1BAJH-1526-SM-7RHGI | Liver | above60 |
| GTEX-1EKGG-1226-SM-7IGNO | Liver | above60 |
| GTEX-S33H-1626-SM-4AD68 | Liver | below40 |
| GTEX-148VI-0626-SM-5TDDH | Liver | above60 |
| GTEX-17F96-1226-SM-79OK2 | Liver | above60 |
| GTEX-18A66-2026-SM-7189C | Liver | above60 |
| GTEX-1QCLZ-1226-SM-EAZ49 | Liver | above60 |
| GTEX-X4EO-1126-SM-4QARQ | Liver | above60 |
| GTEX-1GF9V-1226-SM-7MKGY | Liver | above60 |
| GTEX-15DYW-1326-SM-6LPIV | Liver | above60 |
| GTEX-1I1HK-1126-SM-AHZ2L | Liver | above60 |
| GTEX-REY6-1226-SM-48FDR | Liver | above60 |
| GTEX-S4Z8-0526-SM-4AD4T | Liver | above60 |
| GTEX-1EWIQ-0726-SM-7MKFW | Liver | above60 |
| GTEX-11TUW-1726-SM-5BC5C | Liver | above60 |
| GTEX-13PVR-0126-SM-5S2PY | Liver | above60 |
| GTEX-11OF3-0726-SM-5BC4Z | Liver | above60 |
| GTEX-12ZZZ-1326-SM-59HKW | Liver | above60 |
| GTEX-UTHO-2426-SM-4JBHD | Liver | above60 |
| GTEX-1ICG6-1026-SM-B2LY6 | Liver | above60 |
| GTEX-1GN73-1126-SM-9OSW5 | Liver | above60 |
| GTEX-1JJEA-1526-SM-CNNPK | Liver | above60 |
| GTEX-14PJO-1726-SM-68719 | Liver | above60 |
| GTEX-11DXY-0526-SM-5EGGQ | Liver | above60 |
| GTEX-QESD-2026-SM-447BI | Liver | below40 |
| GTEX-1JJ6O-0826-SM-CXZJL | Liver | above60 |
| GTEX-U8XE-1526-SM-4E3HT | Liver | below40 |
| GTEX-1IKK5-0326-SM-C1YPY | Liver | above60 |
| GTEX-13QJC-0726-SM-5RQJK | Liver | above60 |
| GTEX-14E7W-1526-SM-6871X | Liver | above60 |
| GTEX-1KWVE-0326-SM-EV7AF | Liver | below40 |
| GTEX-ZTPG-1426-SM-51MT3 | Liver | below40 |
| GTEX-11TT1-1726-SM-5EQLJ | Liver | below40 |
| GTEX-14753-1626-SM-5NQ9L | Liver | above60 |
| GTEX-YECK-1926-SM-4W21H | Liver | above60 |
| GTEX-OOBJ-0826-SM-3NB2K | Liver | above60 |
| GTEX-SJXC-1226-SM-4DM78 | Liver | above60 |
| GTEX-18A6Q-2026-SM-718AQ | Liver | above60 |
| GTEX-11EQ9-0526-SM-5A5JZ | Liver | below40 |
| GTEX-13NZB-0626-SM-5IFH6 | Liver | below40 |
| GTEX-1212Z-0226-SM-59HLF | Liver | above60 |
| GTEX-ZVT3-1626-SM-5GU66 | Liver | above60 |
| GTEX-1GF9W-0826-SM-7MKHI | Liver | above60 |
| GTEX-O5YV-0726-SM-EV798 | Liver | above60 |
| GTEX-1A8G7-1126-SM-731ED | Liver | above60 |
| GTEX-14XAO-0226-SM-68728 | Liver | above60 |
| GTEX-Y5V5-0926-SM-4VBPZ | Liver | above60 |
| GTEX-1B8L1-0726-SM-7P8RT | Liver | above60 |
| GTEX-12696-0826-SM-5EGGE | Liver | above60 |
| GTEX-OIZF-0826-SM-3MJGO | Liver | above60 |
| GTEX-ZYY3-0626-SM-5NQ6W | Liver | above60 |
| GTEX-1AX9I-1626-SM-72D5D | Liver | above60 |
| GTEX-131YS-1626-SM-5HL6C | Liver | above60 |
| GTEX-1B996-1426-SM-7EPIA | Liver | above60 |
| GTEX-144GM-1326-SM-5LU5E | Liver | below40 |
| GTEX-12WSM-0726-SM-5GCOW | Liver | above60 |
| GTEX-12KS4-1326-SM-5LUB3 | Liver | below40 |
| GTEX-11NV4-1326-SM-5HL6V | Liver | above60 |
| GTEX-PX3G-0826-SM-48TZS | Liver | below40 |
| GTEX-O5YT-0826-SM-3TW8N | Liver | below40 |
| GTEX-1EH9U-1126-SM-7IGLU | Liver | above60 |
| GTEX-P44G-1126-SM-3NM9D | Liver | below40 |
| GTEX-1QPFJ-1126-SM-E9U5V | Liver | below40 |
| GTEX-1PBJJ-0626-SM-DPRYO | Liver | below40 |
| GTEX-1NV8Z-1626-SM-DPRYT | Liver | above60 |
| GTEX-1399R-1226-SM-5P9GF | Liver | below40 |
| GTEX-QEG4-1826-SM-CKZN9 | Liver | below40 |
| GTEX-147GR-1326-SM-7IGLB | Liver | above60 |
| GTEX-13113-1326-SM-5GCOI | Liver | above60 |
| GTEX-ZF29-2026-SM-DNZYW | Liver | above60 |
| GTEX-14DAQ-1726-SM-5S2R2 | Liver | above60 |
| GTEX-1CB4F-1726-SM-7MGXH | Liver | above60 |

**Suppl. Table 6.** PCR primers used

| **Custom designed** | **Forward sequence** | **Reverse sequence** |
| --- | --- | --- |
| Catalase | GCTCCGCAATCCTACACCAT | GGACATCGGGTTTCTGAGGG |
| Col1a1 | GGAGAGAGCATGACCGATGG | AAGTTCCGGTGTGACTCGTG |
| CTGF | CTGACCTAGAGGAAAACATT | AGAAAGCTCAAACTTGACAG |
| Fibronectin | AAGACAGATGAGCTTCCCCA | TGGTTCGCCTAAAGCCATGT |
| IL-6 | AAGTCGGAGGCTTAATTACATATGTTC | TCAGAATTGCCATTGCACAAC |
| LOX1 | CCTCTTCCATGGGCCCTTTA | GCCCCTGGTCCTAAAGAATTG |
| Myh 6 | CCCTATAAGTGGCTGCCAGTGT | GTACTGATAGGCGTTGTCAGAGATG |
| NOX2 | CTGCCAGTGTGTCGGAATCT | TGTGAATGGCCGTGTGAAGT |
| TGF-beta1 | GCTGCTGACCCCCACTGAT | GCCACTGCCGGACAACTC |
| TNF-alpha | TCCCTCAGGGGTGTCCTTAG | GTCGTAGCAAACCACCAAGC |
| VCAM | GAGGGTCTACCAGCTCCTGA | AACAGTAAATGGTTTCTCTTGAACA |
| **Commercially available from Qiagen** | | **Catalog number** |
| Beta actin | | QT00193473 |
| Beta-2-microglobulin | | QT00176295 |
| CD68 | | QT00372204 |
| F4/80 | | QT00391272 |
| HPRT | | QT00199640 |
| Myh 7 | | QT00189504 |
| NOX1 | | QT00403501 |
| NOX3 | | QT01575728 |
| Rplp0 | | QT00365561 |

Table lists the PCR primers used. CD68: cluster of differentiation 68; Col1a1: collagen 1a1; CTGF: connective tissue growth factor; HPRT: Hypoxanthine Phosphoribosyltransferase 1; IL-6: interleukin-6; LOX1: oxidized low density lipoprotein 1 receptor; Myh 6: alpha myosin heavy chain; Myh 7: beta myosin heavy chain: NOX 1, 2, 3: NADPH oxidase 1, 2, 3; Rplp0: Ribosomal Protein Lateral Stalk Subunit P0; TGF-beta1: Transforming Growth Factor Beta 1; TNF-alpha: tumor necrosis factor; VCAM: vascular cell adhesion molecule 1.

**Suppl. Fig. 1.** Results of Single cell and spatial transcriptomics

(A) The Uniform Manifold Approximation and Projection for Dimension Reduction (UMAP) plot showing the expression of PCSK9 and (B) TNNT2 (Troponin T, a cardiomyocyte marker) based on the single-cell transcriptomics data from the Heart Cell Atlas (<https://heartcellatlas.org>). (C) PCSK9 is only expressed in less than 0.07%, while (D) TNNT2 is expressed in almost all of the ventricular cardiomyocyte cells. (E) The average Unique Molecular Identifier (UMI) levels of PCSK9 is less than 0.0006 ± 0.0001 (mean±SEM) while (F) the average TNNT2 UMI level is 7.85 ± 0.03. (G) The spatial transcriptomics data also showed that the PCSK9 level in heart is very low compared to (H) TNNT2.

**
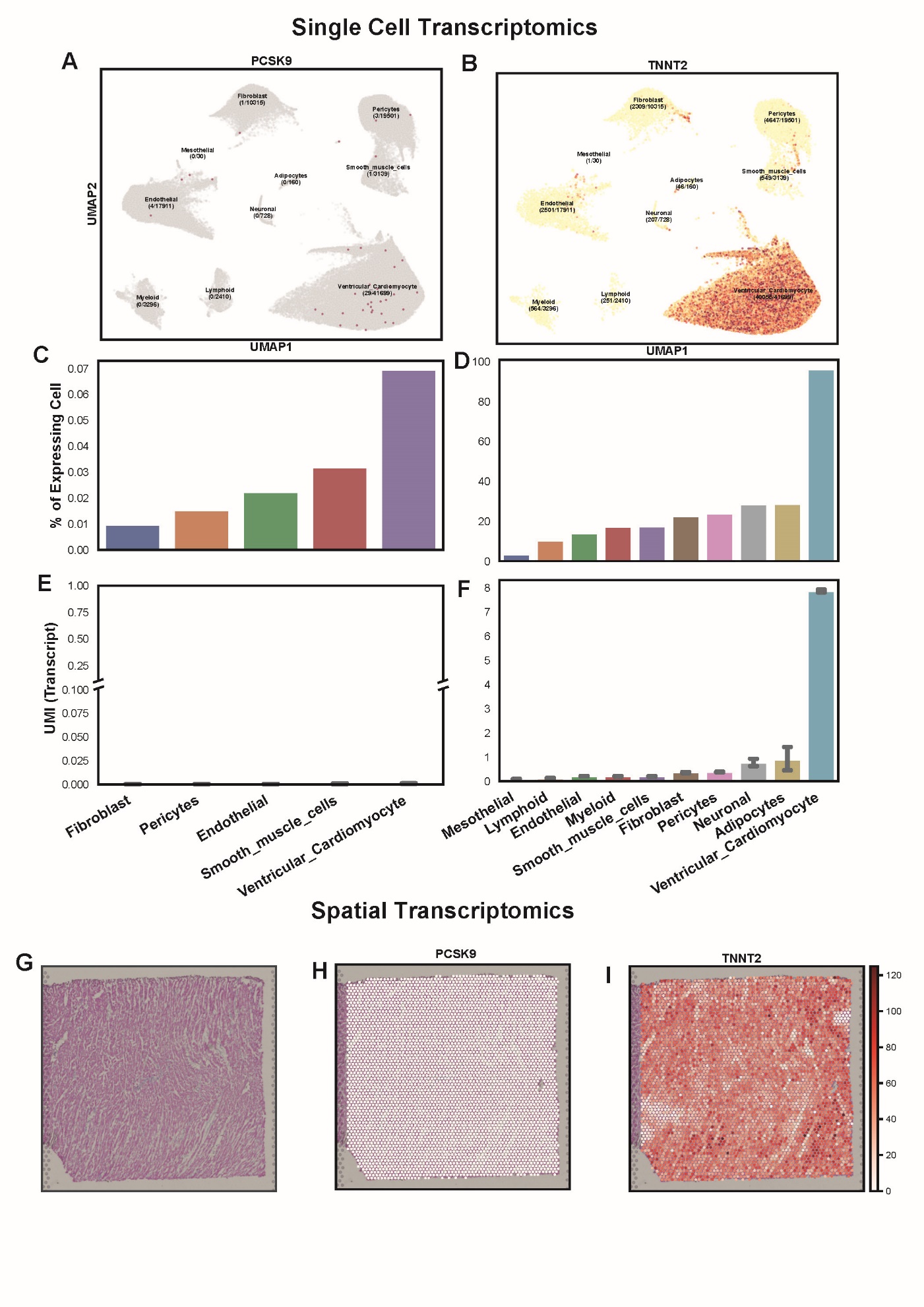
**

**Suppl. Fig. 2.** Serum markers of liver injury

Aspartate and alanine transaminase (AST, ALT) serum levels in the rat model. Scattered dot plots and mean±SEM. Statistics: One-way ANOVA with Tukey’s post-hoc test for multiple pairwise comparisons.

**Suppl. Fig. 3.** Transcriptomic analysis – results of KEGG pathways and GO CC and BP terms

The following analyses were used to show selected pathways relevant in aging, cardiac function and mitochondria on Figure 7.

A.Uniform Manifold Approximation and Projection (UMAP) of cardiac samples


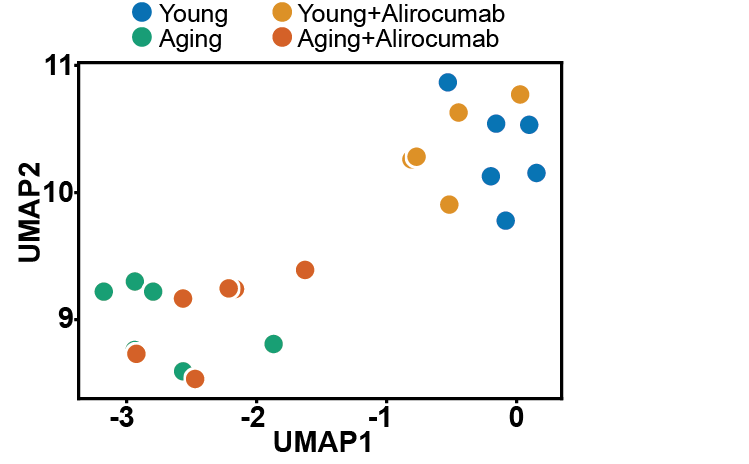


B. Differential expression of genes from transcriptomic analysis. Abbreviations: aging (A), aging+alirocumab (AT), young (Y), young+alirocumab (YT).
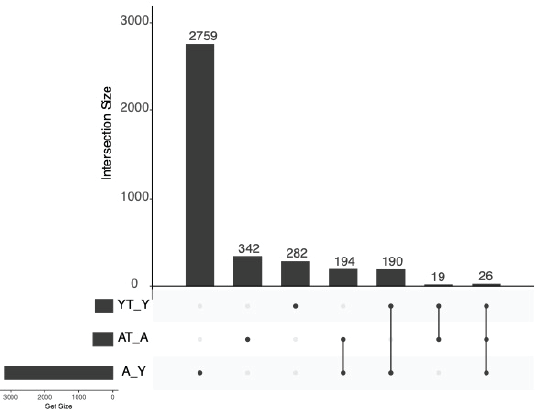


C. Selected KEGG pathways related to Aging.


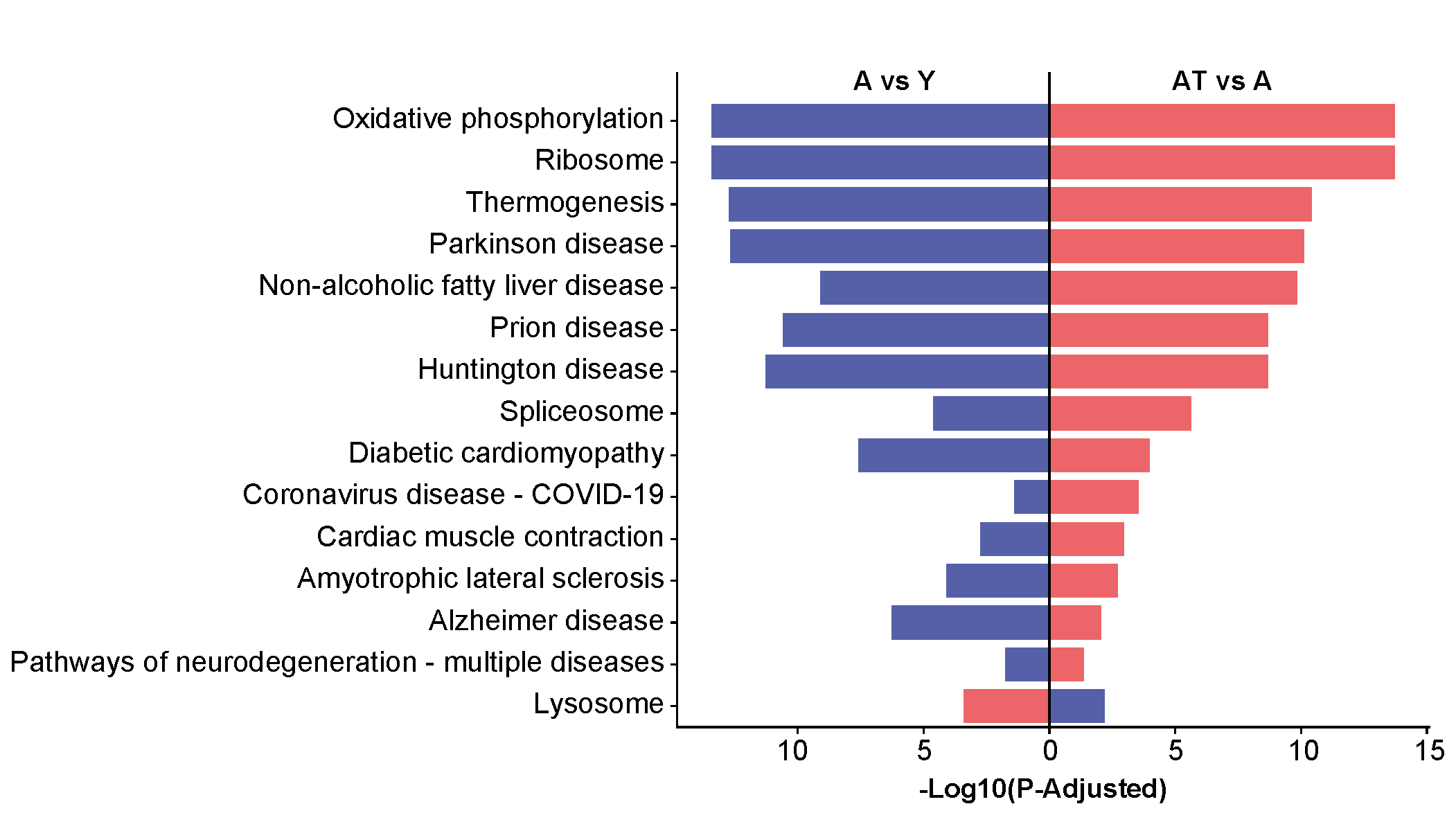


D. Selected GO CCs related to Aging


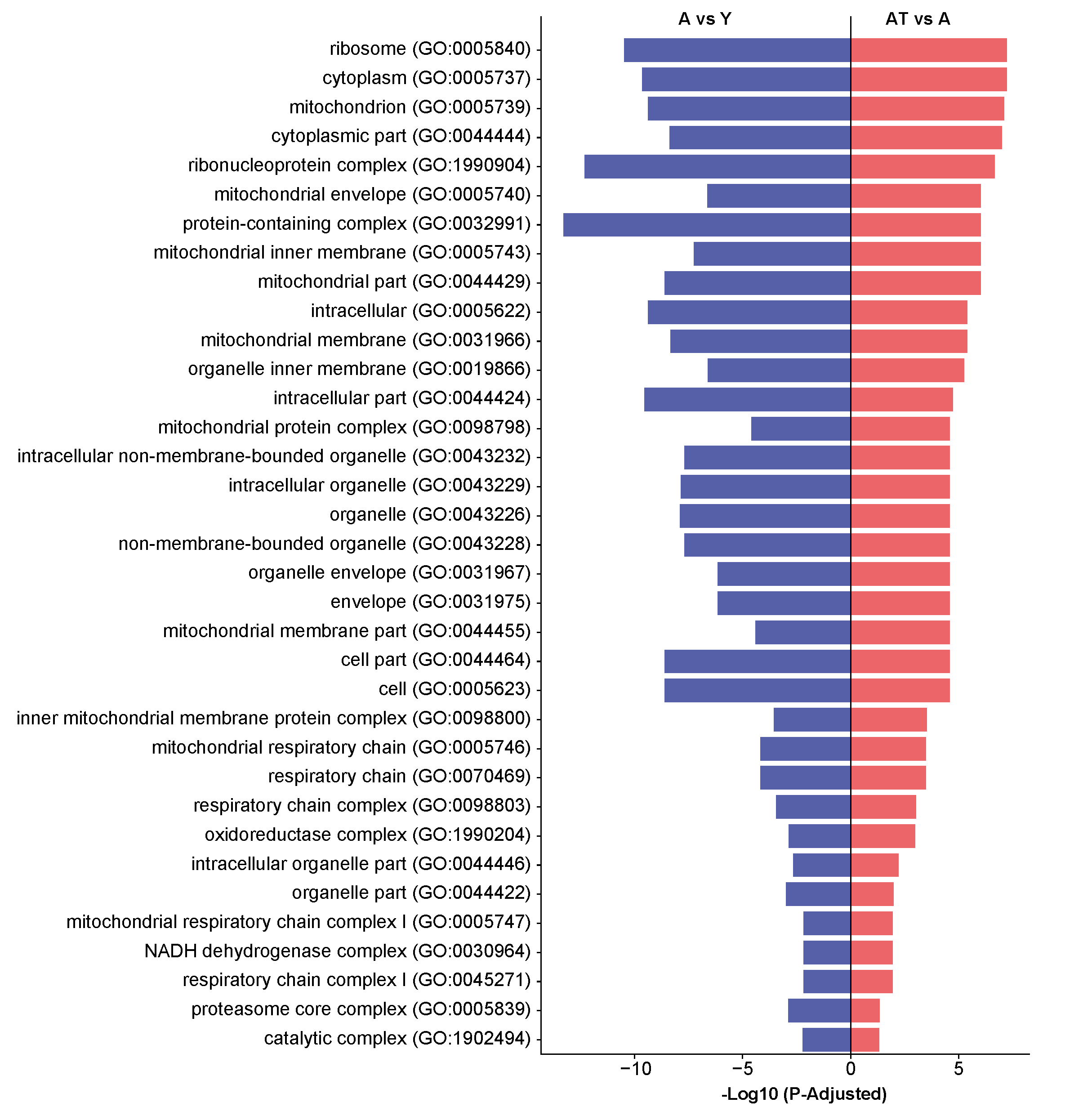


E. Selected GO BPs related to Aging

**
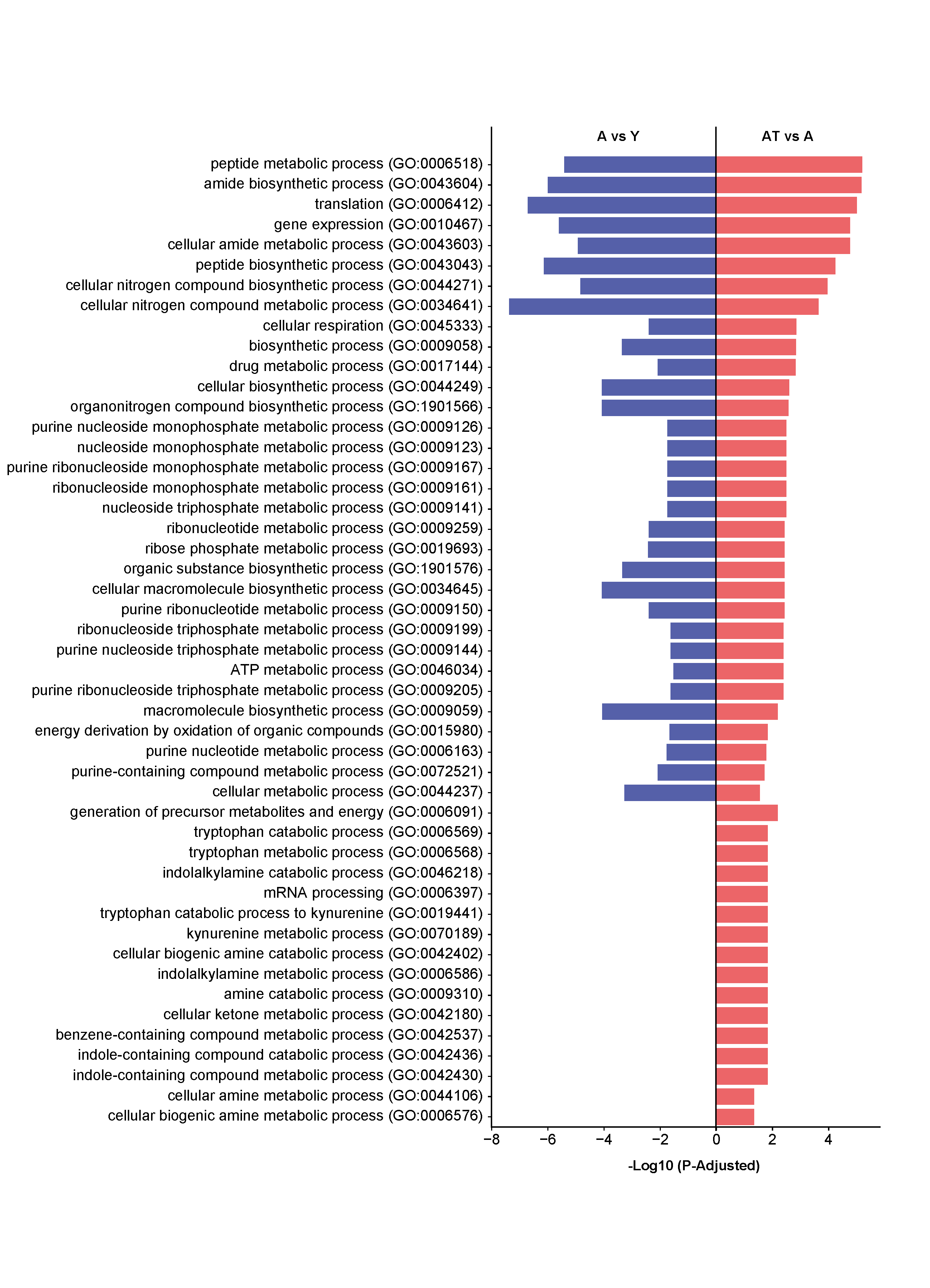
**

**Suppl. Fig. 4.** Protein levels of mitochondrial complexes

A. Protein level of mitochondrial complex I, II, III, IV, V. Scattered dot plots and mean±SEM.Statistics: One-way ANOVA with Tukey’s post-hoc test for multiple pairwise comparisons. *p<0.05 vs Young

B. Representative western blot of mitochondrial complexes


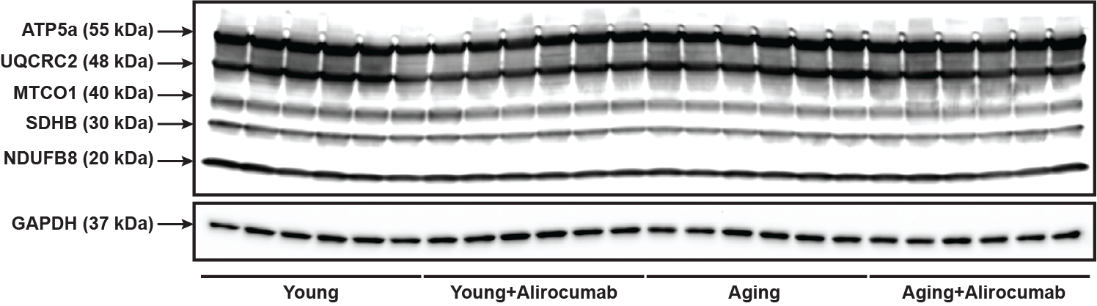

Supplement: Supplemental Appendix [file mmc1.docx]
